# Dysfunctional peripheral T follicular helper cells dominate in people with impaired influenza vaccine responses: Results from the FLORAH study

**DOI:** 10.1371/journal.pbio.3000257

**Published:** 2019-05-17

**Authors:** Suresh Pallikkuth, Lesley R. de Armas, Stefano Rinaldi, Varghese K. George, Li Pan, Kristopher L. Arheart, Rajendra Pahwa, Savita Pahwa

**Affiliations:** 1 Department of Microbiology and Immunology, University of Miami Miller School of Medicine, Miami, Florida, United States of America; 2 Department of Epidemiology and Public Health, Division of Biostatistics, University of Miami Miller School of Medicine, Miami, Florida, United States of America; Weatherall Institute of Molecular Medicine, UNITED KINGDOM

## Abstract

Antigen-primed cluster of differentiation (CD) 4^+^ T follicular helper (Tfh) cells interact with B cells in the germinal centers (GCs) of lymph nodes to generate vaccine-induced antibody (Ab) responses. In the circulation, peripheral Tfh (pTfh) cells, a subset of memory CD4 T cells, serve as surrogates for GC Tfh because of several functional and phenotypic similarities between them. We investigated features of H1N1 influenza antigen-specific pTfh (Ag.pTfh) in virologically controlled HIV^+^ volunteers on antiretroviral therapy (ART) and healthy control (HC) participants selected from a seasonal influenza vaccine responsiveness study. Selection of the participants was made based on age, defined as young (18–40 y) and old (>60 y) and on their classification as a vaccine responder (VR) or vaccine nonresponder (VNR). VRs demonstrated expansion of CD40L^+^ and CD69^+^ Ag.pTfh, with induction of intracellular interleukin 21 (IL-21) and inducible costimulator (ICOS) post vaccination; these responses were strongest in young HC VRs and were less prominent in HIV^+^ individuals of all ages. Ag.pTfh in VNRs exhibited dramatically different characteristics from VRs, displaying an altered phenotype and a cytokine profile dominated by cytokines IL-2, tumor necrosis factor alpha (TNF-α), or IL-17 but lacking in IL-21. In coculture experiments, sorted pTfh did not support the B cell IgG production in VNRs and were predominantly an inflammatory T helper 1 (Th1)/T helper 17 (Th17) phenotype with lower ICOS and higher programmed cell death protein 1 (PD1) expression. Induction of IL-21 and ICOS on Ag.pTfh cells are negatively affected by both aging and HIV infection. Our findings demonstrate that dysfunctional Ag.pTfh cells with an altered IL-21/IL-2 axis contribute to inadequate vaccine responses. Approaches for targeting inflammation or expanding functional Tfh may improve vaccine responses in healthy aging and those aging with HIV infection.

## Abstract 2

This study identifies qualities of flu vaccine-specific T follicular helper cells that are important for vaccine responses, and delineates deficiencies that are associated with impaired vaccine responses in biologic aging and HIV infection in humans.

## Introduction

Decline in immune function has been well described in the setting of physiologic aging [1–6] manifesting as impaired vaccine responses and diminution of antibody (Ab)-secreting cells [7,8] with reduced numbers of lymph node germinal centers (GCs) [9]. CD4 T cells provide help to antigen-primed B cells to undergo proliferation, isotype switching, and somatic hypermutation resulting in the generation of long-lived plasma cells and memory B cells (MBCs; reviewed in [10]). This help is mediated by a specialized CD4 T-cell subset known as T follicular helper (Tfh) cells, characterized by the expression of the lymphoid follicle homing receptor CXC chemokine receptor 5 (CXCR5), which is required for the cells to migrate to the GC. We and others have described a circulating counterpart of CXCR5^+^ Tfh cells known as peripheral Tfh (pTfh) cells that are easily accessible from peripheral blood samples and are able to induce B cell differentiation [11–16]. Studies in healthy adults have documented the importance of pTfh expansion at day 7 or day 28 post vaccination for their association with influenza vaccine response [17–22]. However, most of the reported studies of pTfh (defined as CXCR5^+^ memory CD4 T cells) describe the kinetics of expansion in either bulk pTfh or their phenotypic subsets, providing limited information about antigen reactivity, especially in the aging population and those aging with HIV infection. The phenotypic identity of the bulk pTfh cells in previous flu vaccine studies were highly variable between different labs, making it difficult to draw conclusive evidence related to their identity, specificity, and functionality [18–20].

Influenza vaccination is recommended in individuals considered to be at increased risk for influenza infection, including adults older than 65 y and all HIV-infected persons even if they are virologically suppressed on antiretroviral therapy (ART) [23]. Declining immunity against influenza vaccines has also been reported in HIV-uninfected elderly populations [24–26]. An intriguing feature of HIV infection is that the immune system is believed to undergo premature immune senescence [27–29]. Moreover, HIV+ persons of older age can have worse immune deficits than occurring as a natural consequence of aging [8,30,31]. This has gained added importance because the success of ART has led to rapid growth of virologically suppressed aging HIV^+^ individuals, with adults >50 y expected to exceed 70% of all HIV/AIDS cases by 2020 [29,32]. Thus, it is crucial to better understand the impact of HIV infection on the age-associated decline in immunity. We have demonstrated a decrease in immune response to influenza vaccines in HIV^+^ menopausal women compared to uninfected age-matched healthy controls [8] as well as in HIV^+^ males and females of varying ages [14,33].

In order to understand the Ab response to influenza vaccine and the effect of aging with or without HIV infection, we conducted the present study in young and old HIV^+^ and HIV^-^uninfected (healthy control [HC]) participants who had already been classified as vaccine responders (VRs) and vaccine nonresponders (VNRs) based on their serologic responses to seasonal influenza vaccine [33,34]. We focused on antigen-specific pTfh (Ag.pTfh) identified on the basis of antigen-induced up-regulation of molecules CD40L and CD69 rather than bulk pTfh of unknown specificities [35,36]. Studies of functional attributes of Ag.pTfh cells with respect to VR and VNR status in the context of flu vaccination in healthy and HIV aging are not known. In this study, ex vivo quantitative and qualitative assessment of Ag.pTfh revealed key features of Ag.pTfh that favored vaccine responsiveness. In VRs, magnitude of response was impacted by both quality and quantity of Ag.pTfh cells, and these were compromised in old age in HCs and in young and old HIV^+^ individuals. In VNRs, in contrast, Ag.pTfh were heavily weighted towards an inflammatory phenotype irrespective of age or HIV status. This is the first study in humans to report an association between Ag.pTfh dysfunction and VNR status in aging and in ART-controlled HIV^+^ individuals. The insight gained by this study of Ag.pTfh in peripheral blood provides direction for identifying therapeutic targets for improving vaccine responses.

## Results

**Fig 1:**
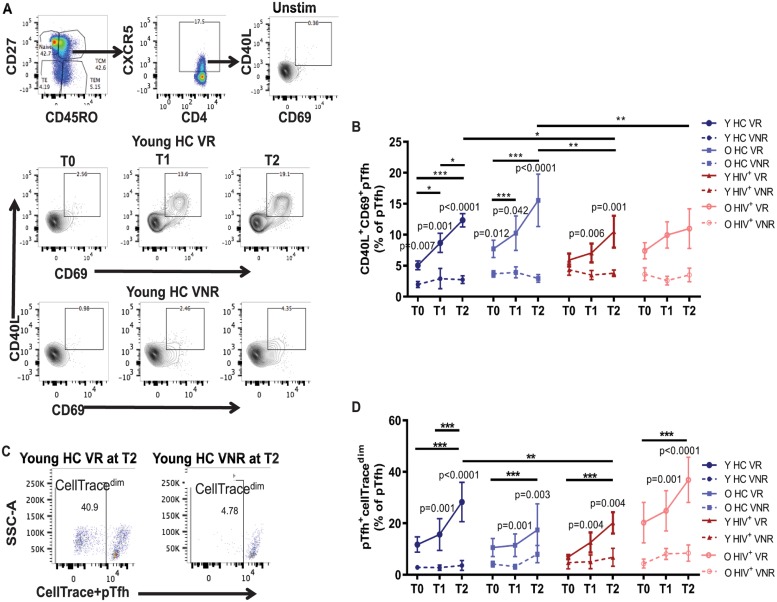
Ag.pTfh expand following influenza vaccination in VRs. H1N1 Ag.pTfh cells were identified by flow cytometry after 12 h of PBMC stimulation with H1N1 antigen in influenza-vaccinated young and old HIV^+^ individuals (red) and HCs (blue) at baseline (T0), day 7 (T1), and day 28 (T2) post vaccination. Ag.pTfh cells were defined as memory (CD45RO^+^CD27^+^) CXCR5^+^CD40L^+^CD69^+^ CD4 T cells. (A) Flow cytometry dot plots showing gating strategy for Ag.pTfh cells at T0, T1, and T2 in a young HC VR and VNR. (B) Line graph showing mean frequencies of Ag.pTfh cells for the indicated participant groups. Error bars indicate SEM. (C) Flow cytometry dot plots showing proliferating (CellTrace^dim^) pTfh cells (CD45RO^+^CD27^+^CXCR5^+^CD4^+^) in young HC VRs and VNRs at T2 following culture with H1N1 antigen (5 d). (D) Line graph showing mean frequencies of CellTrace^dim^pTfh cells for the indicated participant groups with error bars indicating SEM. Statistical analysis was performed to compare study groups and longitudinal analyses using generalized linear mixed models. Horizontal lines indicate significant differences between time points within a group and between groups, and the level of significance corresponds to *p*-values as follows: *p < 0.05; **p < 0.01; ***p < 0.001. *p*-Values shown within the graph refer to significant difference between VRs and VNRs at indicated time points. Underlying data used in the generation of this figure can be found in S1 Data. Ag.pTfh, antigen-specific peripheral T follicular helper; CD, cluster of differentiation; CXCR5, CXC chemokine receptor 5; HC, healthy control; O HC, old healthy control; O HIV^+^, old HIV+; PBMC, peripheral blood mononuclear cell; Unstim, unstimulated cultures; VNR, vaccine nonresponder; VR, vaccine responder; Y HC, young healthy control; Y HIV^+^, young HIV+.

Following ex vivo stimulation of peripheral blood mononuclear cells (PBMCs) with H1N1 antigen for 12 h, Ag.pTfh with dual expression of CD40L and CD69 were clearly discernible within bulk pTfh cells defined as CXCR5^+^ memory CD4 T cells (CD45RO^+^CD27^+^) (Fig 1A). Based on the variables of age (Young versus Old), HIV status (HC versus HIV^+^), and vaccine response status (VR versus VNR), participants were divided into the following 8 groups: (1) young HC VRs, (2) young HC VNRs, (3) old HC VRs, (4) old HC VNRs, (5) young HIV^+^ VRs, (6) young HIV^+^ VNRs, (7) old HIV^+^ VRs, and (8) old HIV^+^ VNRs. Each group was investigated for quantitative and qualitative Ag.pTfh characterization at prevaccination (T0) and at 2 time points post vaccination (T1 and T2), with the goal of determining how VRs differ from VNRs.

### VNRs exhibit lower frequencies of Ag.pTfh with failed expansion and proliferation post vaccination

In HCs, frequencies of Ag.pTfh in young and old VRs increased from T0 to T1 and T0 to T2 and were greater than the corresponding VNR groups at all 3 time points but were not different from one another (Fig 1B and S1 Fig). At T2, frequencies of Ag.pTfh in HCs correlated with the fold change (FC) H1N1 Ab titer at T2 (Table 1). In HIV^+^ young and old VRs, the increases of Ag.pTfh from T0 to T1 and T2 were not significant, but the frequencies were higher than VNRs at T1 and T2 in young, but not in old, HIV^+^ individuals. Frequencies of Ag.pTfh at T0 were not different between young and old HIV^+^ persons, and expansion post vaccination in young HIV^+^ VRs at T2 was significantly less than in young and old HCs. Old HIV^+^ VRs showed a trend of greater frequencies of Ag.pTfh compared to old HIV^+^ VNRs, but it did not reach significance at post-vaccination (Fig 1B). Frequencies of Ag.pTfh at T2 in HIV^+^ individuals failed to show a correlation with FC in H1N1 Ab titer at T2 (Table 1). VNRs in all groups showed similar Ag.pTfh frequencies, indicating that the failure to expand Ag.pTfh post vaccination is a characteristic feature of VNRs regardless of age or HIV status.

**Table 1:** Correlations between Ag.pTfh characteristics at T2 with FC Ab titer.

Ag.pTfh characteristics at T2	HC		HIV	
	r	*p*	r	*p*
Ag.pTfh (%)	0.4	0.006	0.13	NS
pTfh^+^CellTrace^dim^ (%)	0.46	0.007	0.39	0.006
Il-21^+^Ag.pTfh (%)	0.56	<0.0001	0.15	NS
ICOS^+^Ag.pTfh (%)	0.37	0.006	−0.04	NS
IL-2^+^Ag.pTfh (%)	−0.51	0.0001	−0.31	0.028
TNFα ^+^Ag.pTfh (%)	−0.4	0.002	−0.4	0.004
IL-17^+^Ag.pTfh (%)	−0.6	<0.0001	−0.35	0.011

Spearman correlation was performed for correlation analysis, and *p* < 0.05 was considered significant. Correlation analysis included data from young and old VRs and VNRs together for HCs and HIV^+^ individuals.

**Abbreviations**: Ab, antibody; Ag.pTfh, antigen-specific peripheral T follicular helper; FC, fold change; HC, healthy control; ICOS, inducible costimulator; IL-21, interleukin 21; NS, not significant; TNFα, tumor necrosis factor alpha; VNR, vaccine nonresponder; VR, vaccine responder.

To confirm H1N1-specific pTfh cell expansion, we measured in vitro pTfh proliferation following H1N1 antigen stimulation in PBMCs using the CellTrace dye dilution assay. Examples of the proliferating CellTrace^dim^ pTfh cells in young HC VRs and VNRs are shown in Fig 1C. In agreement with the observed increase in ex vivo Ag.pTfh frequencies shown in Fig 1B, an increase in the frequency of proliferating pTfh cells at T2 in comparison to T0 was observed in both young and old HC VRs. Additionally, the frequencies of CellTrace^dim^ pTfh cells in HC VRs (young and old) at T1 and T2 were higher than the corresponding HC VNRs (Fig 1D and S1 Fig). The same results were obtained in the HIV^+^ VRs with CellTrace^dim^ pTfh cells with the exception that the peak proliferating cells in HIV^+^ young VRs was reduced relative to HC young VRs (Fig 1D and S1 Fig). Frequencies of proliferating pTfh cells at T2 directly correlated with FC H1N1 Ab titer at T2 in both HC and HIV^+^ individuals (Table 1). H1N1 antigen-induced pTfh proliferation was absent in VNR groups irrespective of age or HIV status.

### H1N1-induced interleukin 21 and inducible costimulator expression are impaired in Ag.pTfh from HIV^+^ participants

**Fig 2:**
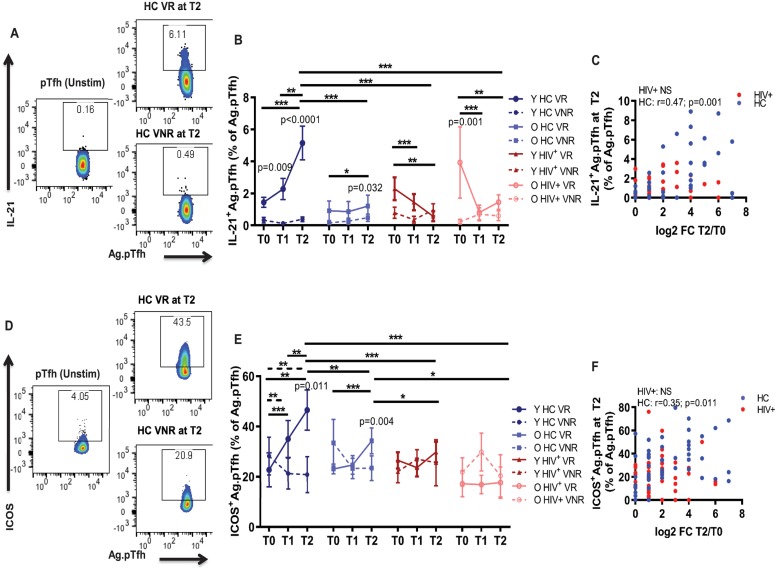
IL-21 and ICOS induction in Ag.pTfh cells are impaired in HIV^+^ groups. (A) Representative flow cytometry dot plots showing IL-21 expression within Ag.pTfh cells in a young HC VR and VNR at T2 post vaccination. (B) Line graph showing mean frequencies of IL-21^+^Ag.pTfh cells in the study groups. (C) Correlation between IL-21^+^Ag.pTfh cells at T2 and FC H1N1 Ab titer at T2 including VRs and VNRs together for HCs (blue) and HIV ^+^ individuals (red). (D) Representative flow cytometry plots showing ICOS^+^Ag.pTfh cells in a young HC VR and VNR. (E) Line graph showing mean frequencies of ICOS^+^Ag.pTfh cells in the study groups. (F) Correlation between ICOS^+^Ag.pTfh cells at T2 with FC H1N1 Ab titer at T2 including VRs and VNRs together for HCs (blue) and HIV^+^ individuals (red). Statistical analysis was performed to compare study groups and longitudinal analyses using generalized linear mixed models. Horizontal lines (solid for VR, dashed for VNR) indicate significant differences between time points within a group and between groups and the level of significance corresponds to *p*-values as follows: *p < 0.05; **p < 0.01; ***p < 0.001. *p*-Values shown within the graph refer to significant difference between VRs and VNRs at indicated time points. For correlation analyses, Spearman correlation coefficient was performed. Underlying data used in the generation of this figure can be found in S1 Data. Ab, antibody; Ag.pTfh, antigen-specific peripheral T follicular helper; FC, fold change; HC, healthy control; ICOS, inducible costimulator; IL-21, interleukin 21; O HC, old healthy control; O HIV^+^, old HIV+; Unstim, unstimulated cultures; VNR, vaccine nonresponder; VR, vaccine responder; Y HC, young healthy control; Y HIV^+^, young HIV+.

To explore the qualitative characteristics of Ag.pTfh pre and post vaccination, we examined their ability to produce the Tfh signature cytokine interleukin 21 (IL-21) [10,37,38] following ex vivo stimulation of PBMCs with H1N1 antigen (Fig 2A). In young HC VRs, an increase in the frequencies of IL-21^+^Ag.pTfh was observed over time from T0 to T2, and frequencies were significantly higher than in young HC VNRs at T1 and T2. Moreover, at T2, the frequencies of IL-21^+^Ag.pTfh cells in young HC VRs were greater than those in the VRs of all 3 other groups at T2. In old HC VRs, frequencies of IL-21^+^Ag.pTfh showed an increase from T0 to T2, and frequencies were higher than the old HC VNRs (Fig 2B and S2 Fig). A completely different pattern was observed in the HIV^+^ VR group that was marked by a decrease in frequencies of IL-21^+^Ag.pTfh over time from T0 to T2 in both young and old (Fig 2B and S2 Fig). At T2, IL-21^+^Ag.pTfh directly correlated with FC H1N1 Ab titer in HC only (Fig 2C and Table 1). VNRs from all groups demonstrated a failure of IL-21 production by Ag.pTfh regardless of age or HIV status. Our data indicate that in HCs, IL-21 production by Ag.pTfh is an important determinant for the magnitude of Ab response post vaccination, and this feature is disrupted in virologically controlled HIV^+^ vaccine recipients of all ages.

Given the importance of inducible costimulator ligand (ICOS-L) interactions during T cell-dependent Ab production [10,39,40], we analyzed surface expression of ICOS on Ag.pTfh cells (Fig 2D). While T0 ICOS expression was not different among any of the groups (mean 20%–30%), ICOS induction in young HC VRs after vaccination followed a similar pattern as IL-21 and was induced maximally at T2, at which time point it was greater than that at T2 in all 3 other groups. ICOS was induced to a lower magnitude in old HC VRs, and not at all in HIV^+^ young and old VRs (Fig 2E and S2 Fig). Moreover, VNRs from all groups failed to increase ICOS expression on Ag.pTfh post vaccination irrespective of age or HIV status (Fig 2E and S2 Fig). At T2, ICOS^+^Ag.pTfh directly correlated with FC H1N1 Ab titer in HC only (Fig 2F). Furthermore, ICOS^+^Ag.pTfh at T0 (S2 Fig) and at T2 (S2 Fig) directly correlated with IL-21^+^Ag.pTfh at T2 only in HCs. Taken together, these data indicate a striking impairment within young and old HIV^+^ individuals in ICOS and IL-21 induction in Ag.pTfh cells that is not directly associated with magnitude of Ab response following influenza vaccination. Although a causal relationship between induction of ICOS and IL-21 on Ag.pTfh with Ab response to vaccination was not proven, a lower expression of the two was associated with lower magnitude of Ab responses following vaccination in HCs.

### VNRs produce higher Tfh antagonistic and inflammatory cytokines post vaccination

**Fig 3:**
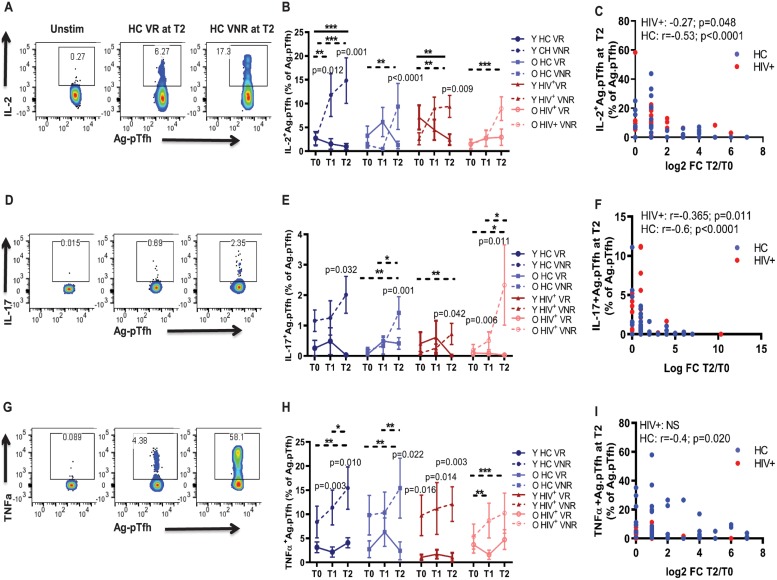
Ag.pTfh in VNRs showed higher production of Tfh antagonistic and inflammatory cytokines at T2. (A); Flow cytometry plots showing IL-2^+^Ag.pTfh cells in a young HC VR and VNR. (B) Line graph showing mean frequencies of IL-2^+^pTfh cells in the study groups. (C) Correlation between IL-2^+^Ag.pTfh cells at T2 with FC H1N1 Ab titer at T2 including VRs and VNRs together for HCs (blue) and HIV^+^ individuals (red). (D) Flow cytometry dot plots showing IL-17^+^Ag.pTfh cells in a young HC VR and VNR. (E) Line graphs showing mean frequencies of IL-17^+^Ag.pTfh. (F) Correlation between IL-17^+^Ag.pTfh cells at T2 with FC H1N1 Ab titer at T2. (G) Flow cytometry dot plots showing TNFα^+^Ag.pTfh cells in a young HC VR and VNR. (H) Line graphs showing mean frequencies of TNFα^+^Ag.pTfh. (I) Correlation between TNFα^+^Ag.pTfh cells at T2 with FC H1N1 Ab titer at T2. Statistical analysis was performed to compare study groups and longitudinal analyses using generalized linear mixed models. Horizontal lines (solid for VR, dashed for VNR) indicate significant differences between time points within a group and between groups and the level of significance corresponds to *p*-values as follows: *p < 0.05; **p < 0.01; ***p < 0.001. *p*-Values shown within the graph refer to significant difference between VRs and VNRs at indicated time points. For correlation analyses, Spearman correlation coefficient was performed. Underlying data used in the generation of this figure can be found in S1 Data. Ab, antibody; Ag.pTfh, antigen-specific peripheral T follicular helper; FC, fold change; HC, healthy control; IL, interleukin; O HC, old healthy control; O HIV^+^, old HIV+; Tfh, T follicular helper; TNFα, tumor necrosis factor alpha; Unstim, unstimulated cultures; VNR, vaccine nonresponder; VR, vaccine responder; Y HC, young healthy control; Y HIV^+^, young HIV+.

To investigate the mechanisms of Ag.pTfh dysfunction in VNRs, we measured induction of additional cytokines, including IL-2, IL-17, and tumor necrosis factor alpha (TNF-α) (Fig 3A, 3D and 3G). Frequencies of Ag.pTfh producing IL-2 (Fig 3B and S2 Fig), IL-17 (Fig 3E and S2 Fig), and TNF-α (Fig 3H and S2 Fig) increased in VNRs from T0 to T2 in young and old HCs and young HIV^+^ individuals and were greater than respective VRs at 1 or more time points. In the old HIV^+^ VNR group, the percentage of Ag.pTfh producing IL-2 and TNF-α at T2 was not significantly different than VRs but did show a significant increase at T2 compared to T0. IL-2 has been shown to antagonize Tfh differentiation [41–43], and our data show that in young HCs and young HIV^+^ VRs, IL-2 levels decreased significantly from T0 to T2 (Fig 3B). Baseline expression levels of these cytokines were not different between the study groups, showing that the observed effects were a result of influenza vaccination. Furthermore, T2 expression of all 3 cytokines had a negative correlation with FC H1N1 Ab titer in HCs, while IL-2 and IL-17 were negatively correlated with FC H1N1 Ab titer in HIV^+^ individuals (Fig 3C, 3F and 3I). A strong positive correlation was found between TNF-α ^+^Ag.pTfh and IL-2^+^Ag.pTfh at T2 in both HCs and HIV^+^ individuals (S2 Fig). Analysis of the PBMC culture supernatants from proliferation experiments showed higher levels of inflammatory cytokines, IL-17 (S3 Fig) and TNF-α (S3 Fig), at T0 and T2 in both, HIV^+^s and HC VNRs compared to the respective VR groups, along with a lower production in VNRs of activin A, a protein that promotes Tfh differentiation [44] (S3 Fig).

To further understand the nature of pTfh polarization in vivo, we performed Boolean gating and Simplified Presentation of Incredibly Complex Evaluations (SPICE; version 5.3, Vaccine Research Center, NIAID, US) analysis of cytokines induced by influenza peptides in pTfh of VRs and VNRs. We performed this analysis in CD40L^+^ pTfh cells instead of CD40L^+^CD69^+^ pTfh cells because we found too few pTfh cells with co-expression of CD40L and CD69 in some VNR participants, especially in the HIV^+^ VNR groups. A representative SPICE graphical output in a young HC at T0, T1, and T2 is shown in S4 Fig. Single IL-21-expressing CD40L^+^ pTfh cells were greater in VRs compared to VNRs in all the groups and were highest in young HCs, increasing from T0 to T2 (S4 Fig). CD40L^+^ pTfh expressing TNF-α either alone (S4 Fig) or in combination with IL-2 (S4 Fig) or IL-21 (S4 Fig) were higher in VNRs from all groups, suggesting that IL-21 production in the absence of pro-inflammatory and Tfh-antagonizing cytokines is important for optimal vaccine responses.

### Frequency and function of Ag.pTfh cells correlates with MBC phenotype

We have recently reported on the B cell phenotypic and functional characteristics in young and old HCs and HIV^+^ persons at T0 in these study participants [33]. Here, we investigated the association between Ag.pTfh cells with frequency and function of H1N1-specific Ab-producing MBCs enumerated by ELISpot assay at T2. H1N1-specific MBCs at T2 were present in higher frequencies only in HCs, correlated with IL-21^+^Ag.pTfh cells (S5 Fig), and were inversely correlated with TNF-α^+^Ag.pTfh cells at T2 (S5 Fig). Resting MBCs at T2 directly correlated with Ag.pTfh cells in both HCs and HIV^+^ individuals at T2 (S5 Fig), indicating an association between Ag.pTfh and resting memory (RM) B cell development post vaccination.

### Baseline immune activation in bulk pTfh cells is detrimental to Ag.pTfh frequency and ICOS induction post vaccination

**Fig 4:**
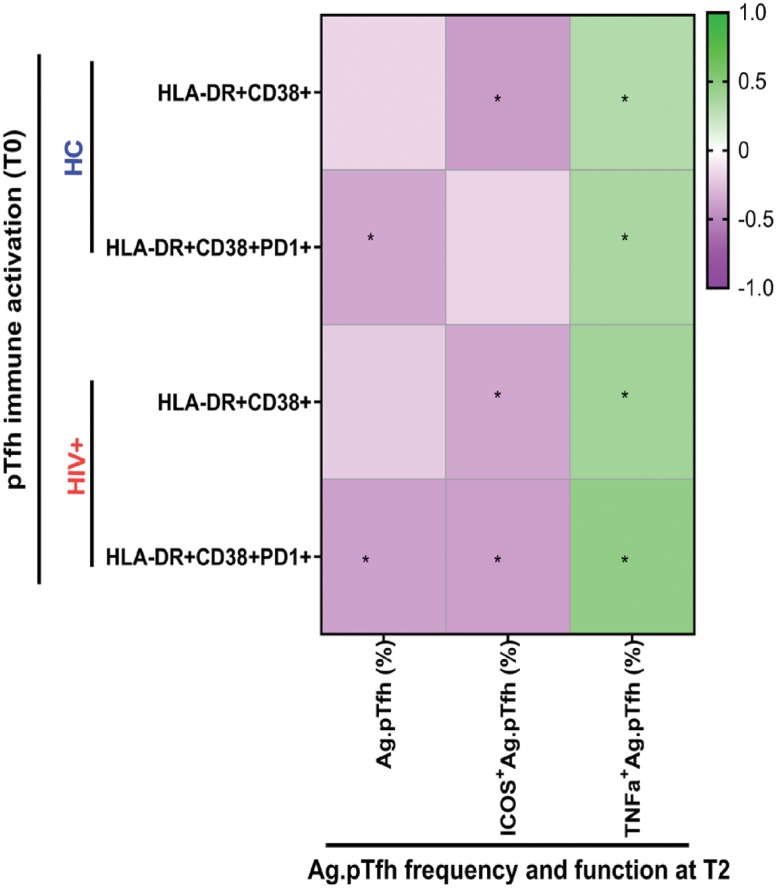
Association of Ag.pTfh frequency and functional markers with baseline immune activation of pTfh cells. Heatmap showing correlation coefficients between immune activation parameters in bulk pTfh cells at T0 with Ag.pTfh characteristics, including frequency, ICOS expression, and TNFα production at T2. All correlation analysis were performed by including VRs and VNRs together for HC (blue) and HIV ^+^ (red) groups. Colored boxes represent correlation coefficient for each comparison, and scale indicates positive r value (green) and negative r values (purple). For correlation analyses, either Pearson or Spearman rank correlation coefficient was performed based on data distribution. *p < 0.05 indicates significant correlations (see also S1 Table). Underlying data used in the generation of this figure can be found in S1 Data. Ag.pTfh, antigen-specific peripheral T follicular helper; HC, healthy control; ICOS, inducible costimulator; pTfh, peripheral T follicular helper; TNFα, tumor necrosis factor alpha; VNR, vaccine nonresponder; VR, vaccine responder.

We recently reported a negative association between activated pTfh at T0 and influenza vaccine response in this cohort [34]. We used co-expression of CD38 and Human Leukocyte Antigen–DR isotype (HLA-DR) (with or without programmed cell death protein 1 [PD1]) on pTfh cells as a measure of immune activation and evaluated whether the basal state of activation in pTfh influenced Ag.pTfh characteristics at T2 (Fig 4). Data for the correlation coefficient (r) and *p*-values are shown in S1 Table. In both HCs and HIV^+^ individuals, frequencies of activated (HLA-DR^+^CD38^+^) pTfh cells at T0 correlated directly with TNF-α^+^Ag.pTfh and inversely with frequencies of ICOS^+^Ag.pTfh at T2. Moreover, PD1 expressing activated pTfh at T0 directly correlated with TNFα^+^Ag.pTfh and inversely with frequencies of Ag.pTfh at T2 in HCs and HIV^+^ individuals and ICOS^+^Ag.pTfh in HIV^+^ individuals. However, Ag.pTfh expressing IL-21, IL-2, and IL-17 at T2 did not show correlations with baseline immune activation of pTfh cells in HCs and HIV^+^ individuals (S2 Table). These data support baseline immune activation as a potential mechanism for reduced Ag.pTfh frequencies and impaired ICOS induction on Ag.pTfh in some influenza vaccine recipients.

### Key variables associated with influenza Ab titer response in HCs and HIV^+^ individuals

**Fig 5:**
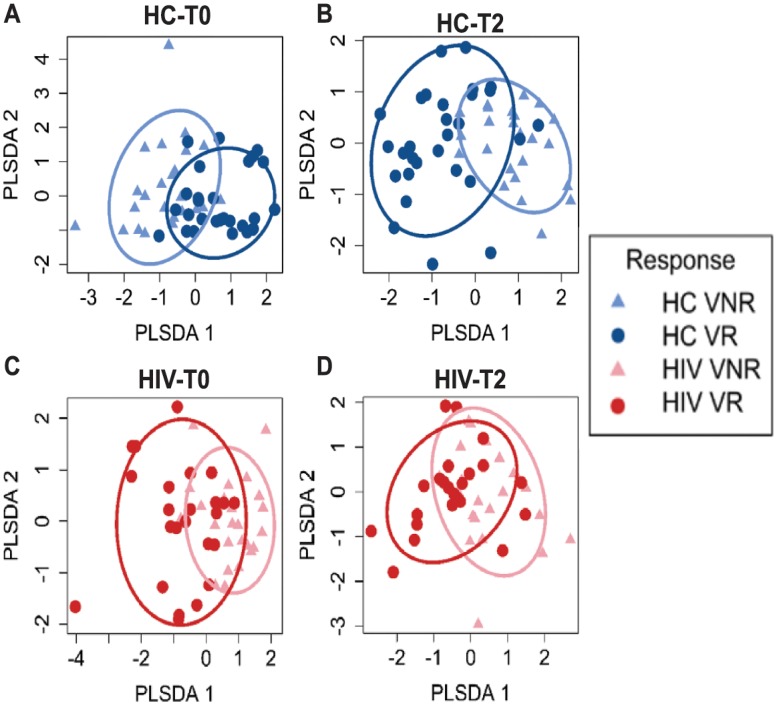
Identification of Ag.pTfh variables at T0 and T2 that are associated with FC H1N1 Ab titer at T2 in HCs and HIV^+^ individuals. LASSO and PLSDA identified 4 variables at T0 and 3 variables at T2 for HC (blue) and 3 variables at T0 and 2 variables at T2 for HIV^+^ individuals (red), which were selected for their association with FC H1N1 Ab titer shown in Tables 2, 3, 4 and 5. Underlying data used in the generation of this figure can be found in S1 Data. Ab, antibody; Ag.pTfh, antigen-specific peripheral T follicular helper; FC, fold change; HC, healthy control; LASSO, least absolute shrinkage and selection operator; PLSDA, partial least squares discriminant analysis; VNR, vaccine nonresponder; VR, vaccine responder.

In our univariate correlation analysis between the T2 Ag.pTfh properties and FC H1N1 Ab titer in the HC and HIV^+^ individuals, we found significant associations in HCs (Table 1), but in HIV^+^ individuals, associations of FC H1N1 Ab titer and Ag.pTfh frequency and key molecules such as IL-21 and ICOS were lacking. In order to identify the most important variables for Ab response in each group, we used the statistical method least absolute shrinkage and selection operator (LASSO) [45] and partial least squares discriminant analysis (PLSDA). Twenty-six antigen-specific measures—including those identified after in vitro stimulation of PBMCs with H1N1 antigen—and bulk pTfh data collected longitudinally at T0 and T2 were entered as x-variables for LASSO analysis using the FC H1N1 Ab titer as the outcome (S2 Table). This analysis generated “predictive” models containing variables that were able to segregate VRs and VNRs in HCs (Fig 5A) and HIV^+^ individuals (Fig 5C) at T0 as well as “correlative” models with variables that could segregate VRs and VNRs at T2 (Fig 5B and 5D). For HCs, 4 variables at T0 and 3 variables at T2 were selected by LASSO for their association with FC H1N1 Ab titer (Tables 2 and 3). At T0, frequencies of Ag.pTfh and IL-21^+^Ag.pTfh had a positive association, whereas IL-17^+^ and IL-2^+^ Ag.pTfh cells were negatively associated with FC H1N1 Ab titer at T2. At T2, frequencies of Ag.pTfh, IL-21^+^Ag.pTfh, and CD40L^+^CD69^+^ CD4 T cells were selected for their association with FC H1N1 Ab titer. For HIV^+^ individuals, 3 variables at T0 (frequencies of Ag.pTfh and IL-21^+^Ag.pTfh and proliferating [CellTrace^dim^] pTfh) directly associated with the FC H1N1 Ab titer. At T2, two variables (Tables 4 and 5)—namely, proliferating (CellTrace^dim^) pTfh and CD40L^+^ pTfh—were selected for their association with FC H1N1 Ab titer. These data concur with the univariate linear regression analysis supporting the specific roles of frequencies of Ag.pTfh and IL-21^+^Ag.pTfh as predictive biomarkers of vaccine responsiveness in both HCs and HIV^+^ individuals, as well as immune correlates of vaccine-induced response in HCs, which is impaired in HIV^+^ individuals. None of the immune activation biomarkers from T0 were selected for the model, indicating that functional analyses of Ag.pTfh is a more robust measurement for identifying correlates of response to influenza vaccination.

**Table 2:** Variables associated with FC (T0) H1N1 response in HCs at T0.

T0	Estimate	Std. Error	t value	Pr(>|t|)
(Intercept)	2.04726	0.5749	3.5606	0.0008
Ag.pTfh (% of pTfh)	0.8835	0.2220	3.9788	0.0002
IL-21^+^Ag.pTfh (% of Ag.pTfh)	0.5476	0.2355	2.3245	0.0244
IL17^+^ pTfh (% of CD40L + pTfh)	−0.6254	0.2364	−2.6455	0.01105
IL2^+^Ag.pTfh (% of Ag.pTfh)	−0.52357	0.2111	−2.4796	0.0167

Residual standard error: 1.287 on 47 DF; multiple R-squared: 0.5109; adjusted R-squared: 0.4589; F-statistic: 9.819 on 5 and 47 DF, *p*-value: 1.8 × 10^−06^; α = 0.8; λ = LambdaMin.

**Abbreviations**: Ag.pTfh, antigen-specific peripheral T follicular helper; CD, cluster of differentiation; FC, fold change; HC, healthy control; IL-21, interleukin 21.

**Table 3:** Variables associated with FC (T2) H1N1 response in HCs at T2.

T2	Estimate	Std. Error	t value	Pr(>|t|)
(Intercept)	−3.3487	1.2130	−2.7606	0.0080
Ag.pTfh (% of pTfh)	1.2413	0.3280	3.7839	0.0004
IL21^+^Ag.pTfh (% of Ag.pTfh)	0.6114	0.1546	3.9545	0.0002
CD40L^+^CD69^+^ CD4 (% of CD4)	0.6050	0.1969	3.0716	0.0034

Residual standard error: 1.21 on 49 DF; multiple R-squared: 0.5494, adjusted R-squared: 0.5218; F-statistic: 19.92 on 3 and 49 DF, *p*-value: 1.405 × 10^−08^; α = 1; λ = Lambda1SE

**Abbreviations**: Ag.pTfh, antigen-specific peripheral T follicular helper; CD, cluster of differentiation; FC, fold change; HC, healthy control; IL-21, interleukin 21.

**Table 4:** Variables associated with FC (T0) H1N1 response in HIV^+^ individuals at T0.

T0	Estimate	Std. Error	t value	Pr(>|t|)
(Intercept)	−0.2384	0.6222	−0.3831	0.7033
Ag.pTfh (% of pTfh)	0.4063	0.1601	2.5374	0.01461
IL21^+^Ag.pTfh (% of Ag.pTfh)	0.3038	0.1445	2.1021	0.0410
CellTrace^dim^ pTfh (% of pTfh)	0.6162	0.1570	3.9239	0.0002

Residual standard error: 0.9056 on 46 DF; multiple R-squared: 0.4789, adjusted R-squared: 0.4449; F-statistic: 14.09 on 3 and 46 DF, *p*-value: 1.2 × 10^−06^; α = 1; λ = Lambda1SE.

**Abbreviations**: Ag.pTfh, antigen-specific peripheral T follicular helper; CD, cluster of differentiation; FC, fold change; IL-21, interleukin 21.

**Table 5:** Variables associated with FC (T2) H1N1 response in HIV^+^ individuals at T2.

T2	Estimate	Std. Error	t value	Pr(>|t|)
(Intercept)	−0.4310	0.7574	−0.5690	0.5720
CellTrace^dim^ pTfh (% of pTfh)	0.6692	0.1596	4.1912	0.0001
CD40L^+^pTfh (% of pTfh)	0.5362	0.1936	2.7696	0.0080

Residual standard error: 0.9883 on 47 DF; multiple R-squared: 0.3658, adjusted R-squared: 0.3389; F-statistic: 13.56 on 2 and 47 DF, *p*-value: 2.247 × 10^−05^; α = 1; λ = LambdaMin.

**Abbreviations**: CD, cluster of differentiation; FC, fold change; IL-21, interleukin 21; pTfh, peripheral T follicular helper.

### Ex vivo IL-21 supplementation along with IL-2 and TNF neutralization during pTfh:B cell interaction partially rescues Ag.pTfh defects and improves B cell function in VNRs

**Fig 6:**
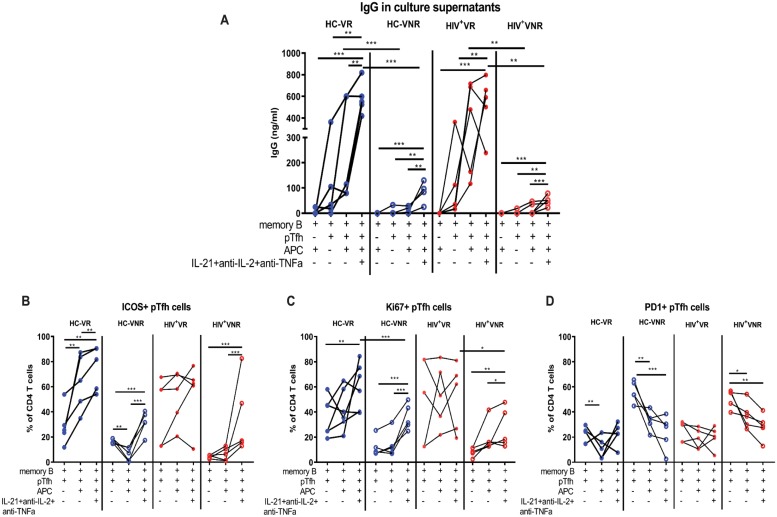
Ex vivo IL-21 supplementation along with IL-2 and TNF-α neutralization during pTfh:B cell interaction improves B cell function in VNRs. Cryopreserved PBMCs obtained at T2 post vaccination were thawed and rested overnight. pTfh (Aqua^−^CD19^-^CD3^+^CD8^-^CD4^+^CD45RO^+^CXCR5^+^), MBCs (Aqua^−^CD3^−^CD19^+^CD27^+^) and APC (Aqua^−^CD3^−^CD19^−^CD8^−^) population were purified by cell sorting. B cells were cocultured with pTfh and APC at a 1:1:1 ratio in medium alone or in the presence of 5 μg/mL of H1N1 vaccine Ag +/− a cytokine cocktail containing IL-21 (50 ng/ml), anti-IL-2 (10 μg/ml) and antiTNF-α (2 μg/ml) for 7 d. (A) Culture supernatants were harvested on day 7, and IgG production was measured by ELISA. (B–D) Cells were harvested and frequency of ICOS^+^, Ki67^+^, and PD1^+^ pTfh cells were evaluated by flow cytometry in the cocultures. For unpaired data, Mann-Whitney U test and for paired data Wilcoxon signed rank test was performed. *p* < 0.05 was considered significant. Line with stars indicates difference between the conditions and groups and the level significance as *p < 0.05; **p < 0.01; ***p < 0.001. Underlying data used in the generation of this figure can be found in S1 Data. Ag, antigen; APC, antigen presenting cell; CXCR5, CXC chemokine receptor 5; ICOS, inducible costimulator; IL-21, interleukin 21; anti-IL-2, antibody against interleukin 2; MBC, memory B cell; PBMC, peripheral blood mononuclear cell; PD1, programmed cell death protein 1; pTfh, peripheral T follicular helper; TNFα, tumor necrosis factor alpha; VNR, vaccine nonresponder.

To further investigate the influence of the cytokine microenvironment on pTfh helper function, we performed coculture experiments using purified pTfh, MBCs, and antigen presenting cells (APCs) collected at T2 from HIV^+^ and HC VRs and VNRs. Cell mixtures were cultured with H1N1 antigen in the presence and absence of a cytokine cocktail containing recombinant IL-21 along with neutralizing Abs against IL-2 (anti-IL-2) and TNF (anti-TNF) and then analyzed for IgG in the supernatant and B and pTfh phenotypes by flow cytometry. B cells cultured with APC alone (without pTfh cells) did not show any IgG production supporting the importance of pTfh cells for optimal B cell function (Fig 6A). In VR groups, pTfh cells were able to support H1N1 antigen-stimulated IgG production by MBCs in the presence of APC, whereas pTfh cells of VNRs failed to do so. These results demonstrate the functional deficiency of pTfh cells that leads to compromised helper function and dampened B cell responses in VNRs. Addition of the cytokine cocktail significantly increased the IgG production in both HCs and HIV^+^ VRs over cells alone. Meanwhile, both HCs and HIV^+^ VNR groups showed significant increases in IgG production in the presence of the cytokine cocktail, albeit with lower levels compared to VRs with or without cytokine cocktail treatment. In order to verify the role of IL-21 in the IgG response in VRs, we performed additional coculture experiments in which sort-purified pTfh, MBCs, and APCs collected at T2 from HCs and HIV^+^ VRs were cultured with H1N1 Ag or *Staphylococcus aureus* enterotoxin B (SEB) in the presence or absence of IL-21 neutralizing Ab. The results show a clear trend of lower IgG production in both H1N1- and SEB-stimulated cultures treated with IL-21 neutralizing Ab in HCs (S6 Fig) and HIV^+^ (S6 Fig) VRs. Taken together, these data support the hypothesis that pTfh cells are functionally impaired in VNRs and suggest that supplementation of IL-21 and neutralization of the effect of IL-2 and TNF during pTfh:B cell interactions may partially rescue the Ab responses in VNRs.

**Fig 7:**
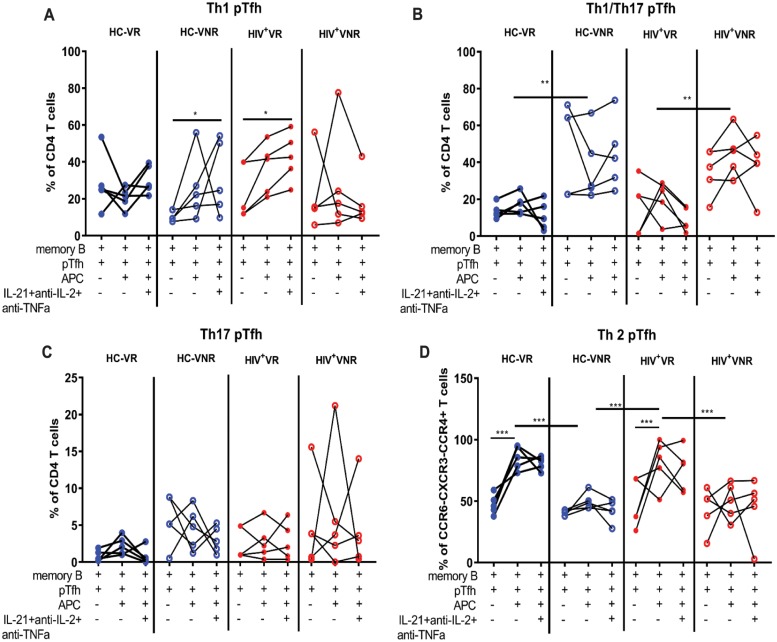
pTfh and MBC differentiation phenotypes in cocultures. PBMCs at T2 were thawed and rested overnight. pTfh (Aqua^−^CD19^−^CD3^+^CD8^−^CD4^+^CD45RO^+^CXCR5^+^), MBCs (Aqua^−^CD3^−^CD19^+^CD27^+^), and APC (Aqua^−^CD3^−^CD19^−^CD8^−^) populations were purified by cell sorting. B cells were cocultured with pTfh and APC at a 1:1:1 ratio in medium alone or in the presence of 5 μg/mL of H1N1 vaccine Ag +/− a cytokine cocktail containing IL-21 (50 ng/ml), anti-IL-2 (10 μg/ml), and anti-TNF-α (2 μg/ml) for 7 d. (A–D) Harvested pTfh were analyzed for T helper differentiation based on CCR4, CCR6, and CXCR3 expression into (A) Th1 (CCR4^−^CCR6^−^CXCR3^+^), (B) Th1/Th17(CCR4^−^CCR6^+^CXCR3^+^), (C) Th17 (CCR4^−^CCR6^+^CXCR3^−^), and (D) Th2 (CCR4^+^CCR6^−^CXCR3^−^) cells. For unpaired data, “Mann-Whitney” U test and for paired data Wilcoxon Signed Rank Test were performed. *p* < 0.05 was considered significant. Line with stars indicates difference between the conditions and groups and the level significance as *p < 0.05; **p < 0.01; ***p < 0.001. Underlying data used in the generation of this figure can be found in S1 Data. Ag, antigen; APC, antigen presenting cell; CCR, CC-chemokine receptor; CXCR, CXC chemokine receptor; IL-2, interleukin 2; MBC, memory B cell; PBMC, peripheral blood mononuclear cell; pTfh, peripheral T follicular helper; Th17, T helper 17; TNFα, tumor necrosis factor alpha.

We also analyzed the phenotypic characteristics of pTfh cells after coculture for the expression of ICOS, Ki67, and PD1. ICOS expression on pTfh cells was strongly up-regulated by the cytokine cocktail treatment in VNRs (Fig 6B). Analysis of Ki67 on pTfh cells showed that pTfh proliferation was higher in both HCs and HIV^+^ VRs compared to VNRs. In the VNRs, the cytokine cocktail resulted in an increase in Ki67^+^ pTfh cells in both HCs and HIV^+^ individuals (Fig 6C). PD1^+^ pTfh cells were higher in the VNRs irrespective of HIV status, with maximum expression in cultures in the absence of APCs (Fig 6D). The cytokine cocktail condition showed a trend of further lowering the PD1 expression on pTfh cells from both HCs and HIV^+^ VNRs. Analysis of Th1, Th2, and Th17 pTfh subsets as defined by the expression of chemokine receptors CC chemokine receptor 4 (CCR4), CCR6, and CXC chemokine receptor 3 (CXCR3) [13] showed a significant increase in frequencies of the Th2 (CCR4^+^CCR6^−^CXCR3^−^) subset in VRs as well as an increase in the Th1/Th17 (CCR4^−^CCR6^+^CXCR3^+^) subset in VNRs (Fig 7A, 7B, 7C and 7D). Analysis of B cells showed higher frequencies of CD19^+^CD20^lo/neg^ B cells and plasmablasts (CD19^+^CD20^lo/neg^CD27^+^CD38^+^) in VRs indicating a higher rate of MBC differentiation in the cocultures of VRs (S7 Fig). In the presence of antiIL-21 Abs, VRs showed a trend of lower frequencies of CD19^+^CD20^lo/neg^ B cells (S7 Fig) and plasmablasts (S7 Fig) indicating the detrimental effect of IL-21 neutralization on MBC differentiation and function. Presence of APC in the cocultures resulted in an increase in the frequencies of Th2 pTfh indicating a Th2 polarization HCs and HIV^+^ VRs (Fig 7D) and an increase in the ICOS expression on pTfh cells in HC VRs (S6 Fig). In VNRs, presence of APC lowers PD1 expression on pTfh cells in both HCs and HIV^+^ individuals (Fig 6D). In the B cell compartment, addition of pTfh to the B cells increases the B cell differentiation compared to B cell:APC cultures in both HC and HIV^+^ VRs. Furthermore, APC:B cell:pTfh cultures further increases B cell differentiation compared to B cell:APC cultures in HCs and HIV^+^ VRs as well as in HC VNRs (S7 Fig). These results suggest that in VNRs, phenotypic and functional alterations of pTfh cells characterized by lower ICOS and higher PD1 expression, may impair their B cell helper function. The cytokine cocktail treatment and the presence of APCs in cocultures could partially rescue these altered phenotypic and functional profiles of pTfh cells in VNRs.

## Discussion

The identification of precise immune correlates of vaccine responses is crucial to the search for biomarkers that predict immune responses to vaccines and for the identification of targets for intervention. We and others have previously shown that the subset of CD4 T cells defined as circulating or pTfh cells are important determinants of immune response to influenza vaccines. In the present study, our goal was to delineate characteristics of Ag.pTfh between established VR and VNR adults in the context of chronologic age and virologically suppressed HIV infection. The antigen-induced up-regulation of molecules CD40L and CD69 on pTfh was used for defining Ag.pTfh cells [46]. We demonstrate that compared to VNRs, Ag.pTfh in VRs are present in higher frequencies pre vaccination and undergo antigen-induced proliferation post vaccination in young and old, with or without HIV. VRs in HCs exhibit a postvaccination increase in ICOS expression with an intracellular cytokine profile dominated by IL-21, and the young VRs are qualitatively superior to old VRs. Compared to HC VRs, levels of IL-21^+^ and ICOS^+^ Ag.pTfh are significantly less in HIV+ VRs, regardless of age. VNRs in all groups manifest a different postvaccination pTfh cytokine profile from VRs, consisting of IL-2, TNF-α, and IL-17. Coculture experiments using purified pTfh, MBCs, and APCs confirmed the functional impairment of pTfh cells in VNRs. In these experiments, although individual cytokine effects were not examined, we verified the beneficial effect of neutralizing cytokines IL-2 plus TNF-α together with exogenous supplementation of IL-21 in improving B cell function during the T cell:B cell interaction. Statistical modelling by LASSO and Elastic Net Regression with input of all immunologic measures in HCs and HIV^+^ individuals revealed overlapping and distinct features impacting the magnitude of Ab response. This study demonstrates attributes of Ag.pTfh that favor or inhibit influenza vaccine responses.

For assessing Ag.pTfh in the context of vaccine-induced immune response, we investigated changes in antigen-induced proliferation and intracellular cytokine expression. Expansion of Ag.pTfh and Ag-induced proliferation post vaccination were evident in VRs in all groups, while VNRs had clear deficiencies in these attributes of Ag.pTfh. The failed expansion of Ag.pTfh cells in VNRs could be attributed, at least in part, to a defect in activin A production. Activin A is a protein recently described to drive Tfh differentiation [44], levels of which were significantly lower in HIN1-stimulated PBMC culture supernatants in VNRs at T0 and T2. Activin A is strongly induced by monocytes during CD40/CD40L-mediated cognate interaction with activated T cells [47].

We examined induction of IL-21 in Ag.pTfh following ex vivo H1N1 stimulation of PBMCs in VRs and VNRs. Among cytokines involved in Tfh function, IL-21 is considered to be the signature cytokine of Tfh [10,38,48–50]. Among VRs, robust IL-21 production by Ag.pTfh at T2 was evident in young HCs and was significantly lower in aging HCs and all HIV^+^ individuals. VNRs of both HCs and HIV^+^ individuals were clearly deficient in induction of IL-21. These observations indicate that aging and HIV infection both lead to impairment of IL-21 production in pTfh cells. Our finding that single IL-21^+^CD40L^+^ pTfh cells predominate in the VR extends recent studies pointing to the importance of circulating IL-21^+^CD4 T cells as biomarkers for vaccine responses [16,51,52]. We observed that the absence of IL-21 production by Ag.pTfh was a striking feature of all VNR groups irrespective of age or HIV status. The presence of IL-21 is an important determinant of the strength of the Ab response in both young and old HC VRs. In HC VRs, IL-21 production tracked with FC in H1N1 Ab titer at T2. Compared to HIV^+^ VNRs, HIV^+^VRs had higher frequencies of preexisting IL-21^+^Ag.pTfh at the time of vaccination (T0) (Fig 2B), similar to the young HC VRs at T0. In contrast to HCs, in whom IL-21^+^Ag.pTfh increased from T0 to T2, the frequencies of IL-21^+^ Ag.pTfh cells in HIV^+^ VRs declined and exhibited limited expansion post vaccination, implying fewer available Ag.pTfh for helping MBCs in the vaccine response. Our LASSO-based analysis for the predictive biomarkers also showed that both frequency of Ag.pTfh and IL-21^+^Ag.pTfh at T0 are important predictive biomarkers for the fold change in Ab response in HIV^+^ individuals from T0 to T2. To further support the role of IL-21 in VRs, we conducted coculture experiments using IL-21-neutralizing Ab in HIV^+^ and HC VRs. Our data showed a trend of lower IgG production in the cocultures in the presence of anti-IL-21 neutralizing Abs in H1N1 and SEB-stimulated cultures from both HIV^+^ and HC VRs, indicating the importance of IL-21 for Ab production by MBCs in cocultures. Taken together, we consider IL-21 to be an important determinant for the strength of the vaccine response in normal, healthy aging and that this is perturbed in HIV infection.

We found that IL-2 production by Ag.pTfh had a negative impact on the influenza Ab response. A VNR status was associated with high IL-2 and low IL-21 production by Ag.pTfh at T2, with a preferential expansion of IL-2^+^Ag.pTfh over IL-21^+^Ag.pTfh cells post vaccination. These findings corroborate our recent observation of differential *IL21/IL2* gene transcription in HIV^+^ children classified as VRs and VNRs, respectively [52]. Higher IL-2 production by Ag.pTfh may antagonize de novo pTfh cell generation and function, as the IL-2 signaling pathway is known to inhibit Tfh cell differentiation through Signal transducer and activator of transcription 5 (STAT5)-mediated expression of *PRDM1* (Blimp-1) [41–43]. The findings reported herein add to the accumulating evidence for the functional significance of the IL-21/IL-2 axis on immunological outcomes [43,44,52–54]. Our data extend a previous report by Cubas and colleagues, in which IL-2-induced detrimental reprogramming in memory bulk pTfh cells resulted in a Th1 polarized phenotype with reduced IL-21 and CXCR5 expression in HIV^+^ aviremic individuals [54]. Our findings indicate that the cytokine profile of the Ag.pTfh compartment can determine the VR and VNR status in the context of flu vaccine response in aging and HIV^+^ populations. Data from coculture experiments indicate that the pTfh differentiation was more towards an inflammatory Th1/Th17 phenotype in VNRs and a Th2 phenotype in VRs during T cell:B cell interaction in both HCs and HIV^+^ individuals. Moreover, addition of anti-IL-2 and anti-TNF-α Abs in the presence of exogenous IL-21 in the cocultures did not alter these differentiation profiles in VNRs and VRs. In the VNRs, the addition of IL-21, along with neutralization of IL-2 and TNF-α, enhanced B cell function, but they remain lower than VRs, possibly due to decreased ICOS expression resulting in inefficient cell-to-cell interaction.

Functionally relevant pTfh cells have been identified by variable phenotypic markers—including PD1, ICOS, and CXCR3 within the memory CXCR5^+^ CD4 T-cell population in HIV infection [16,54]—and also in vaccine studies [8,13,14,18,55]. Notably, CXCR5^+^CXCR3^−^PD1^+^ cells in circulation were reported to be highly functional and correlated with broad neutralizing Ab development in HIV^+^ individuals [12,56]. The flow panel used for phenotypic characterization of the Ag.pTfh in our study did not include PD1 or CXCR3. We rationalized maintaining a broad(er) definition of pTfh cells for achieving our goal of finding novel determinants of influenza vaccine-induced Ab responses in cultures after in vitro stimulation. PD1 is known to be affected by age and by HIV status; we previously observed in the Flu Responses of People in Relation to Age and HIV (FLORAH) cohort, that PD1 expression on CD4 T cells pre vaccination (at T0) correlated with chronologic age and that the PD1^+^ CD4 T cells were higher in both young and old HIV^+^ individuals compared to HCs [34]. In the current study, we observed that PD1-expressing, activated (CD38^+^HLA-DR^+^) total pTfh cells at baseline were inversely correlated with frequencies of Ag.pTfh in both HCs and HIV^+^ individuals, and with ICOS^+^ Ag.pTfh in HIV^+^ individuals. Regarding CXCR3, we utilized the co-expression of CCR6 and CXCR3 to define Th1/Th17 cells in the coculture experiments using sort-purified pTfh cells (CD4^+^CD45RO^+^CD27^+^CXCR5^+^) and found that VNRs had higher frequencies of CXR3+CCR6+ cells. Based on our findings, we posit that both aging and HIV infection may negatively influence the IL-21/IL-2 axis in Ag.pTfh and may thereby impact the vaccine response, possibly by affecting ICOS expression Ag.pTfh.

Induction of ICOS expression in Ag.pTfh cells at T2 was found to be an important determinant of their functional capability, correlated with vaccine responses, and was highest in young HC VRs. ICOS-ICOSL interactions are known to be important for Tfh:B cell interaction and also for *IL-21* gene transcription in Tfh cells through *c-Maf* [57,58]. In HCs, direct correlation between IL-21^+^ Ag.pTfh and ICOS^+^Ag.pTfh indicates the relevance of ICOS in induction of IL-21 in pTfh cells; in HIV^+^ individuals, this association was absent irrespective of age. The association of ICOS expression on pTfh cells and vaccine responses has been established in several recent studies using bulk pTfh cells with varying phenotypes in healthy adults for influenza vaccine [17,18,20] as well as in 3 different human HIV vaccine trials [59]. In fact, a previous study using tetramer staining and activation-induced marker (AIM) assay demonstrated an expansion of the Ag.pTfh population post influenza vaccination, with co-expression of ICOS and CD38 and IL-21 production in young HCs [19]. Here, the ICOS^+^CD38^+^ pTfh correlated strongly with the circulating plasmablast response. However, in our study, we did not include CD38 as a marker of Ag.pTfh, because we found an age-associated decline in CD38 on pTfh cells [34]. Ours is the first study investigating ICOS expression on Ag.pTfh cells in the context of aging and HIV and points to the importance of studying Ag.pTfh compartment because the age-associated defects in ICOS induction post vaccination are not readily apparent in bulk pTfh.

There is accumulating evidence for the deleterious effects of inflammation on immune response [6,60–62], for which the role of IL-2 in promoting TNF-α within activated T cells [63] is especially relevant for this study. Induction of proinflammatory cytokines TNF-α and IL-17 in Ag.pTfh were both increased in VNRs in all groups at T2. Indeed, the increased induction of TNF-α^+^Ag.pTfh cells from VNRs at T2 was negatively correlated with Ab titer and FC T2/T0 response in HCs, and TNF-α^+^Ag.pTfh were strongly correlated with IL-2^+^Ag.pTfh in both HIV^+^ individuals and HCs at T0. Proinflammatory IL-17 has been linked with various autoimmune and inflammatory disorders [64,65] and also with aging in mice [66]. A role of these cells in the production of self-reactive Ab and the formation of dysregulated GCs in autoimmune disease has been established in mouse models [67]. Because HIV infection induces severe depletion of Th17 cells in the gut and impairs the gut integrity [68], further studies are needed to establish the relevance of circulating IL-17^+^Ag.pTfh in the context of influenza vaccination in the aging HIV population.

The deleterious consequences of inflammation were further substantiated by the negative correlation of frequency of Ag.pTfh at T2 with baseline bulk pTfh cells expressing the HLA-DR^+^CD38^+^ activation phenotype [69], as well as expression of PD1. Moreover, baseline immune activation of bulk pTfh correlated directly with TNF-α and negatively with ICOS in Ag.pTfh at T2, indicating that immune activation may promote the generation of pro-inflammatory and qualitatively deficient Ag.pTfh. The detrimental effect of the inflammatory environment on Ag.pTfh cells can be seen by the higher levels of TNF-α and IL-17 in H1N1-stimulated PBMC culture supernatants in VNRs compared to VRs at T0 and T2. These data are in agreement with our previous studies highlighting the detrimental effect of CD4 T-cell immune activation and plasma TNF-α on bulk pTfh frequency post vaccination [8,30,31]. In the B cell compartment, the importance of the quality of Ag.pTfh cells was evident for development of MBC response post vaccination, with IL-21^+^Ag.pTfh cells correlating positively and TNF-α^+^Ag.pTfh correlating negatively with H1N1-specific MBC generation. The importance of IL-21 was further evident from the Boolean analysis of CD40L^+^pTfh cells showing that single IL-21-producing pTfh cells are the predominant population in HCs and HIV^+^ VRs, whereas IL-21 in combination with IL-2 and TNF-α or single TNF-α^+^ or TNF-α^+^IL-2^+^ pTfh cells are higher in VNRs. We also analyzed whether Cytomegalovirus (CMV) serostatus had an influence on Ag.pTfh frequency and function as CMV infection can significantly affect Ab responses to the influenza vaccine in aging and HIV infection [70–73]. In our study groups, CMV serostatus was higher in the HIV^+^ individuals compared to HCs but did not correlate with age or Ab response to flu vaccination [74]. Although we did not measure CMV IgG titer in these participants, CMV serostatus did not show any association with Ag.pTfh frequency and function (not shown).

To further investigate the role of the cytokine microenvironment on pTfh and B cell function, we performed coculture experiments using purified pTfh, MBCs, APCs, and a cocktail containing cytokine IL-21—along with anti-IL-2 and anti-TNF-α neutralizing Abs—to antagonize the effect of exogenous IL-2 and TNF-α during T cell:B cell interactions. The results confirmed the pTfh defects in VNRs and the importance of the ICOS on pTfh cells for their helper function. In VNRs, the B cell function partially recovered in the presence of the cocktail of exogenous IL-21 and neutralizing Abs against IL-2 and TNF-α, increase in ICOS expression, and lowering of PD1 expression on pTfh cells, corroborating the detrimental impact of the IL-2 and TNF-α signaling on molecules associated with pTfh function. In both HCs and HIV^+^ individuals, addition of APCs to the pTfh:B cell cocultures without cytokine cocktail enhanced ICOS expression and lowered PD1 in pTfh in VNRs and enhanced the polarization of Th2 pTfh in VRs. It is interesting to note that in VNRs, addition of APCs to the pTfh:B cell cocultures without cytokine cocktail reduced PD1 expression but failed to increase ICOS expression and IgG production, suggesting a primary role of ICOS over PD1 in enhancing the IgG production in this system. Further studies are needed to understand the pTfh plasticity associated with immune function and signaling events associated with APC:pTfh:B cell interactions that could determine the B cell function in VNRs. These studies are warranted to identify molecules that could be targeted for improving the ICOS signaling and overcoming the detrimental effect of activation induced PD1 signaling on pTfh functions in VNRs.

We used the LASSO model to further identify the antigen-specific T-cell characteristics that have predictive as well as correlative significance for the magnitude of vaccine response in HIV^+^ individuals and HCs. The LASSO method has been used to identify key variables from large, complex datasets contributing to particular outcomes such as protective HIV vaccine Ab profiles [75] and spontaneous control of HIV disease [76]. We identified the predictive potential of the quantity (frequency) and the quality of the Ag.pTfh (IL-21^+^Ag.pTfh) for the magnitude of the vaccine responses in HIV^+^ individuals and HCs. Our results further demonstrate that these markers can also function as immune correlates for the T2 response in HCs. The LASSO analysis strengthens the concept that deficient IL-21 production by Ag.pTfh in both chronologic aging and in HIV infection, along with impaired expansion of Ag.pTfh cells in HIV^+^ individuals, compromise immune function. It is important to note that, although LASSO selected only a few variables due to the stringent selection criteria and strong association, other Ag.pTfh markers (ICOS, TNF-α, and IL-2) identified in the univariate regression analysis are still relevant in the vaccine response in both HCs and HIV^+^ individuals. Taken together, these findings emphasize the important role of H1N1-specific pTfh cells on influenza vaccine-induced H1N1 Ab response.

We conclude that the fundamental immune impairment that may result in VNR status in both HCs and HIV^+^ individuals involves a number of factors: lower frequency of Ag.pTfh pre vaccination, reduced ICOS expression, increased PD1 expression, altered function of Ag.pTfh compartments post vaccination, and expansion of inflammatory TNFα^+^Ag.pTfh and IL-17^+^Ag.pTfh. In addition, poor expansion of Ag.pTfh cells post vaccination in HIV^+^ individuals may further dampen immune function. Our data support the concept of premature immune senescence in the Ag.pTfh compartment in HIV^+^ individuals because the immunological defects in young HIV^+^ individuals were similar to that of old HCs. Our data underscore the need for studies focusing on Ag.pTfh to identify the immunological impairment that may not be detectable in bulk populations. Studies of transcriptomics and cell signaling are warranted to understand the pathways that determine the differentiation of pTfh cells towards IL-2 or IL-21 in HC versus HIV^+^ individuals. These studies could also reveal the mechanisms resulting in ICOS deficiency in the context of age and controlled HIV infection.

## Materials and methods

### Ethics statement

This study was approved by the institutional review boards of the University of Miami (20130399) and Miami Veterans Affairs Medical Center (1160374) and was carried out in accordance with approved guidelines. All the participants were adults, and voluntary written informed consent was obtained from every participant prior to participating in the study, including consent to have their samples stored for future use.

### Study description and participants

Study participants were selected from project FInding Novel Determinants of Flu Responses (FIND), a substudy of FLORAH in which H1N1-specific Ab responses were determined in HIV^+^ individuals and HCs of different ages [74]. Ab response to H1N1/09 vaccine antigen (H1N1 A/California/07/2009) was determined in serum by hemagglutination inhibition (HAI) assay [8,14,31]. Participants with H1N1 Ab titers of ≥1:40 and a ≥4-fold increase at 4 wk post vaccination were classified as VRs as defined by the Food and Drug Administration (FDA), and those who did not meet these criteria were classified as VNRs [77]. The study population of FIND was restricted to participants with a prevaccination H1N1 Ab titer of ≤1:320 to prevent a person from being classified as a VNR due to the presence of a high prevaccination titer and consequent failure to achieve a 4-fold increase in titer post vaccination [74]. Study participants were recruited during the 2013–2014, 2014–2015, and 2015–2016 influenza seasons, with each participant being unique for one of the 3 vaccine seasons.

Characteristics of the study participants are depicted in Table 6. HIV^+^ individuals and HCs were grouped by age into young (<40 y), with 20 HIV^+^ individuals and 18 HCs, and old (≥60 y), with 30 HIV^+^ individuals and 35 HCs. The HIV^+^ participants were on cART with virologic suppression (plasma HIV RNA < 40 copies/ml) for ≥1 y prior to study entry. As established in the parent study, anyone on hormonal replacement therapy, steroids, or immunosuppressant medications or with a diagnosis of active malignancy or immunodeficiency disorder was excluded. CMV seropositivity was higher in the HIV^+^ individuals compared with HCs for young and old age groups, although CMV status did not affect the age or H1N1 Ab response post vaccination [74]. All participants were given a single intramuscular dose of trivalent influenza vaccine (TIV; Seqirus, PA). HIV^+^ individuals were vaccinated as standard of care at the University of Miami and Miami Veteran Affairs Medical Center. Peripheral venous blood was collected pre vaccination (at T0), on day 7 (T1), and on day 21–28 (T2). Serum and plasma were stored at −80 °C, and PBMCs were cryopreserved in liquid N_2_. Absolute CD4 and CD8 T-cell counts, geometric mean H1N1 titer (GMT), and FC H1N1 Ab titer at T2 over T0 (FC) for participants classified as VRs and VNRs in different age groups are shown in Table 6. At T0, the participants were similar in absolute numbers of CD45, CD3, CD4, CD8, CD4/CD8 ratio, and B cell frequencies. The GMT at T2 and FC was highest in young HC VRs compared to all other groups.

**Table 6:** Characteristics of the study participants.

	HIV negative				HIV positive			
	Young		Old		Young		Old	
	VR	VNR	VR	VNR	VR	VNR	VR	VNR
**No. individuals (M/F/Other)**	9 (5/4)	9 (7/2)	20 (12/8)	15 (9/6)	11 (7/4)	9 (6/3)	14 (6/8)	16 (13/2/1)
**Mean age, y (range)**	31.3 (23–39)	31.4 (26–39)	63.7 (60–73)	66.5 (60–74)	30.4 (22–39)	29.6 (19–39)	63.9 (60–72)	66.5 (60–77)
**ART, y (range)**	NA	NA	NA	NA	8.5 (8–9)	6.25 (4–12)	9.88 (1–26)	6.5 (1–16)
**Absolute cell counts, mean ± SD**								
**CD45**	1,901 ± 687	1,804 ± 190	1,261 ± 299	1,775 ± 599	1,616 ± 168	1,574 ± 699	1,514 ± 464	1,848 ± 1,643
**CD8**	523 ± 345	410 ± 216	436 ± 64	306 ± 95	401 ± 334	326 ± 112	295 ± 173	380 ± 237
**CD4**	925 ± 477.9	815 ± 230	1,284 ± 673	640 ± 384	751 ± 583	789 ± 149	658 ± 341	707 ± 792
**CD4/CD8 ratio**	2.5 ± 1.5	2.9 ± 2.4	2.8 ± 1.1	2.5 ± 2.0	2.5 ± 2.5	2.6 ± 0.9	2.4 ± 2.5	2.5 ± 1.4
**B cells (%)**	9 ± 5.6	10.5 ± 6	10.5 ± 5	9.1 ± 7	8.1 ± 4	9.5 ± 5	11.4 ± 6	11 ± 6.6
**GMT of H1N1**								
**T0**	43.2	117.6	54.6	83.8	132.4	160	62.5	83.5
**T1**	296.3	148.1	332.6	100.8*	411.7	235.1	304.5	118.1*
**T2**	1,612.7	201.6*	538.2*	121.3*	823.5*	253.9*	551.6*	128.8*
**FC Ab T2/T0, mean ± SD**	53.3 ± 46.6	1.7±0.4*	25.3±49.5*	1.6±0.5*	8.4±9*	1.6±0.5*	13±19.6*	1.6±0.5*

*Significant compared to young HC VRs.

**Abbreviations**: Ab, antibody; ART, antiretroviral therapy; F, female; FC, fold change; GMT, geometric mean titer; HC, healthy control; M, male; NA; VNR, vaccine nonresponder; VR, vaccine responder.

### Monoclonal antibodies

The following fluorochrome conjugated antihuman monoclonal antibodies (MoAbs) were used for flow cytometry studies: ICOSBV421, ICOSAlexa488, CD40LBV605, CD69BV650, HLA-DRFITC, CD38APCCy7, and TNF-αAPCCy7 from BioLegend (San Diego, CA); CD3BUV395, CD4PerCPCy5.5, CD8Alexa-Fluor700, CCR7PECF594, IL-2BV711, CXCR5Alexa647, IFNγPE-Cy7, PD1BV650, CD45ROAPCH7, CD21PECy5, CD27PerCPCy5.5, IgDFITC, CD10PECy7, and CD20Alexa700 from BD Bioscience (San Jose, CA); IL-21PE, CD27PECy5, and IL-17Alexa488 from e-Biosciences (San Diego, CA); and CD45ROPE-TexasRed from Beckman Coulter (Fullerton, CA). Live/Dead Fixable Aqua Dead Cell Stain Kit and CellTrace Violet Cell Proliferation Kit were from ThermoFisher (Boston, MA). Recombinant human IL-21 (Cat #8879-IL-010) from R&D systems, purified antihuman IL-2 (Cat #3440-ON-500) and antihuman-IL-21 (Cat #MT216G/21.3m) from Mabtech, and antihumanTNF-α (Cat #502922) from BioLegend were used. Samples were acquired on a BD LSRFortessa (BD Biosciences, CA) flow cytometer and analyzed by FlowJo V10 (TreeStar, Inc).

### Ag.pTfh cell frequency and function at T0, T1 and T2

Determination of Ag.pTfh was based on dual expression of CD40L and CD69 as described previously by flow cytometry [35,36,46] on CD4^+^CD45RO^+^CD27^+^CXCR5^+^ cells in H1N1 stimulated PBMCs, and further characterized for intracellular expression of cytokines IL-21, IL-2, TNF-α, and IL-17. Briefly, cryopreserved PBMCs were thawed, rested overnight, washed, suspended at 1.5 million/ml and cultured with both anti-CD28 and anti-CD49d MoAbs at 1μg/ml each and with 5μg/mL H1N1/09 vaccine antigen (gift by Seqirus, PA) at 37°C and 5% CO_2_ for 12 hrs. Cells cultured with only anti-CD28, anti-CD49d as negative controls (medium), and cultured with 1μg/ml SEB (List Biological Labs, CA) served as positive controls. The secretion inhibitor Brefeldin A (10 μg/mL) was added to all cultures for the last 7 h of incubation. Thereafter, cells were washed and surface stained with Live/Dead Aqua for 20 min on ice followed by staining for surface markers CD3, CD4, CD8, CD45RO, CD27, ICOS and CXCR5, fixed, permeabilized and stained for intracellular activation markers CD40L, CD69 and cytokines IL-2, IL-21, IL-17, and TNF- α. Cells were suspended in 1% paraformaldehyde and acquired on a BD LSRFortessa flow cytometer. Ag.pTfh cells were determined as live (Aqua-) CD3^+^CD4^+^ T cells with co-expression of activation markers CD40L and CD69 on CD45RO^+^CD27^+^CXCR5^+^. Expression of intracellular cytokines (IL-2, IL-21, IL-17 and TNF-α) either alone or in combination along with ICOS was determined on the Ag.pTfh. Boolean gate analysis was performed in CD40L+ pTfh cells using the FlowJo platform to identify combinations of 1 or more markers. Samples with a minimum of 100 CD40L+ events in the pTfh gate were used for the Boolean analysis. SPICE (version 5.3, Vaccine Research Center, NIAID) was used to depict the polyfunctional T-cell data obtained after Boolean gating. Frequency of CD40L^+^CD69^+^ pTfh in unstimulated condition was subtracted from values in H1N1 stimulated condition in each individual to get a precise readout for antigen specificity above background levels.

### H1N1 antigen-specific T-cell proliferation at T0, T1 and T2

Thawed PBMCs were rested overnight as described above and labelled with CellTrace violet proliferation tracker dye. 1.5 million/ml CellTrace labelled PBMCs were then incubated with 5μg/mL H1N1/09 vaccine antigen. Cells in medium without any antigen served as negative controls (medium), while cells with SEB at 1μg/ml served as positive controls. Cultures were incubated for 5 d at 37 °C in 5% CO_2_. On day 5, cells were harvested and culture supernatants were stored at -80°C. Cells were stained with Live/Dead Aqua followed by staining for CD3, CD4, CD8, CD45RO, CD27, CXCR5 and ICOS, acquired and analyzed by flow cytometry. Proliferating cells were identified based on the CellTrace dye dilution. Frequencies of H1N1 specific proliferating cells (CellTrace^dim^) were identified within pTfh compartments after and subtracted the values from the unstimulated conditions. Culture supernatants were collected and stored at -80°C.

### Cytokines, activin A, and CXCL13 from PBMC culture supernatants

Cytokines from PBMC culture supernatants were measured using a customized MILLIPLEX Human Cytokine magnetic bead panel (EMD Millipore) or by ELISA following the manufacturer’s instructions [34]. Briefly, culture supernatants were thawed, and centrifuged at 1,000 *g* for 3 min prior to testing. Undiluted culture supernatants were incubated overnight with a mixture of beads specific for IL-2, IL-17, and TNF-α at 4°C with shaking. The mean fluorescence intensity (MFI) data were analyzed with MILLIPLEX Analyst Software V.3.5 (EMD Millipore) and cytokine concentrations were expressed in pg/ml. Activin A (R&D systems) were measured by ELISA and expressed as pg/ml.

### Resting MBCs and H1N1-specific IgG-secreting MBCs at T2

B cell phenotypic subsets were analyzed by flow cytometry using cryopreserved PBMCs as described previously [22,31,33,78]. Briefly, cells were stained with Live/Dead Aqua followed by staining for surface markers and acquired by FC. Total B cells were identified as live (Aqua-) CD3^-^CD20^+^ cells and RM B cells were identified based on the expression of CD10, CD27, CD21 and IgD as CD20^+^CD10^-^IgD^-^CD27^+^CD21^+^. H1N1-specific IgG-secreting MBCs were identified by MBC ELISPOT assay after culturing PBMCs with H1N1 antigen for 5 d as described [8,33].

### Immune activation of bulk pTfh at T0

Thawed PBMCs were rested overnight and stained with Live/Dead Aqua followed by staining for CD3, CD4, CD8, CD45RO, CD27, CCR7, activation (HLA-DR, CD38, PD1), and CXCR5 and acquired by FC [34]. Immune activation was measured on bulk pTfh cells identified as live (Aqua-) CD3^+^CD4^+^CD45RO^+^CD27^+^CCR7^+^CXCR5^+^ based on frequencies of HLA-DR^+^CD38^+^ or HLA-DR^+^CD38^+^PD1^+^ cells.

### Cell sorting

We included 10 HC and 10 HIV^+^ individuals with 5 VRs and 5 VNRs in each group from middle aged, age-matched individuals (age range: 50–60 y) from the FLORAH cohort for this part of the coculture experiments. Cryopreserved PBMCs at T2 were thawed, rested overnight and a three-way sorting was performed to obtain purified pTfh, B and APCs by sterile sorting on a BD FACSAria fusion cell sorter. Briefly, PBMCs were labeled with Live/Dead aqua, CD3, CD4, CD8, CD19, CD27, CD45RO, and CXCR5. pTfh cells were sorted as Aqua^−^CD19^-^CD3^+^CD8^-^CD4^+^CD45RO^+^CXCR5^+^, MBCs were sorted as Aqua^−^CD3^-^CD19^+^CD27^+^ and APC were sorted as Aqua^−^CD3^-^CD19^-^CD8^-^ population. Purity of each sorted population was >97%.

### pTfh:B:APC cocultures

Purified memory B, pTfh, and APCs (1 × 10^4^ cells each) were cocultured at a 1:1:1 ratio in duplicate wells in the presence of 5 μg/mL of H1N1/09 vaccine Ag with 1 μg/mL of anti-CD28 mAb for 7 d in the presence or absence of a cytokine cocktail containing IL-21 (50ng/ml), anti-IL-2 (10 μg/ml) and antiTNF-α (2 μg/ml). Medium alone and B+APC served as negative controls, and 1 μg/mL of SEB served as a positive control. In a subset of HC and HIV^+^ VR participants (n = 3 each), we performed APC:pTfh:B coculture in the presence of H1N1 antigen and SEB +/- antihuman IL-21 neutralizing Ab (10 μg/ml) to test the role of IL-21production by pTfh cells in VRs for the B cell IgG production. Culture supernatants were analyzed for IgG by ELISA. Cells were characterized by flow cytometry for ICOS, PD1, Ki67 and T helper cell polarization phenotypes based on CCR4, CCR6 and CXCR3 expression on pTfh cells. B cells were analyzed for memory phenotype and plasmablasts based CD20, CD19, CD27, CD38 and Ki67 expression.

### Statistical methods

For Ag.pTfh function including proliferation, cells with medium were used as negative controls to identify the background response which was subtracted from the antigen-stimulated cultures to determine the H1N1 induced response. Group and time analyses used generalized linear mixed models to accommodate the repeated measure of time. The first analysis looked at time differences in the outcomes for each group separately. Planned comparisons among the 3 times were made. The other analyses compared the outcomes between 2 different groups at each time. Planned comparisons were made between the 2 groups at each time. For Correlation analyses, Spearman correlation was used assess the strength of the relationships among the outcomes with age and H1N1 titers at each time for different selected groups. The data are presented as scatter plots with regression lines and correlation coefficients with p values. A p value of <0.05 was considered as significant. SAS 9.4 (SAS Institute, Cary, NC) was used for all analyses.

For LASSO analysis, data was log2+1 transformed and univariate linear regression was performed on each independent variable. Feature selection to find a best prediction model was performed using R package glmnet with LASSO or elastic-net regularization. Firstly data was randomly split to train (2/3) and test (1/3) [79]. Then repeated-corrected 10-fold cross validation for glmnet was performed. Variables at both LambdaMin and Lambda1SE were extracted [79]. For continuous outcome variable, fit linear regression was performed on variables selected from either LambdaMin or Lambda1SE. The model with highest adjusted R square and p-value <0.05 was selected as the best model. Hypothesis tests for reliability of the best model were performed using ANOVA (Analysis of Variance) F test. Type I, Type II and Type III Sums of Squares (SS) tests were conducted to test the significance of regression. For discrete outcome variable (Response VNR/VR), PLSDA was performed on variables selected from best model to classify VNR/VR samples using R package mixOmics [80].

## Supporting information

S1 Fig H1N1-specific pTfh cells and pTfh proliferation at T0, T1, and T2. H1N1-specific pTfh cells were identified by flow cytometry in influenza-vaccinated young and old HIV^+^ individuals and HCs at baseline (T0), day 7 (T1), and day 28 (T2) post vaccination after 12 h of PBMC stimulation with H1N1 antigen. Proliferating pTfh cells were identified based on CellTrace dye dilution on day 5 after PBMC stimulation using flow cytometry. (A) Scatter plots showing frequencies of CD40L+CD69+ pTfh (Ag.pTfh) cells between different age groups in HIV^+^ individuals and HCs. (B), Scatter plots showing frequencies CellTrace^dim^pTfh cells. Group and time analyses used generalized linear mixed models to accommodate the repeated measure of time for differences in the outcomes for each group separately between time points and also between 2 different groups at each time. Error lines indicates the mean ± SD. *p* < 0.05 was considered significant. *indicates significant (*p* < 0.05) differences between VR versus VNR at indicated time points, with green star indicating significantly higher in VRs compared to VNRs and lines with stars indicating difference between time points in VRs and VNRs (green line VR; grey line VNR). *p < 0.05; **p < 0.01; ***p < 0.001. Underlying data used in the generation of this figure can be found in S2 Data. Ag.pTfh, antigen-specific peripheral T follicular helper; HC, healthy control; PBMC, peripheral blood mononuclear cell; VNR, vaccine nonresponder; VR, vaccine responder.
(TIF)

S2 Fig H1N1-specific Ag.pTfh cell function and ICOS expression. Scatter plots showing frequencies of (A) IL-21^+^Ag.pTfh cells, (B) ICOS^+^Ag.pTfh cells. (C–D) Correlation between IL-21^+^Ag.pTfh at T2 with ICOS^+^Ag.pTfh (C) at T0 and (D) at T2. Scatter plots showing frequencies of (E) IL-2^+^Ag.pTfh cells, (F) IL-17^+^Ag.pTfh cells, and (G) TNFα^+^Ag.pTfh cells. (H) Correlations between TNFα^+^Ag.pTfh at T2 with IL-2^+^Ag.pTfh at T2. Group and time analyses used generalized linear mixed models to accommodate the repeated measure of time for differences in the outcomes for each group separately between time points and also between 2 different groups at each time. Error lines indicates the mean ± SD. For correlation analyses, Pearson correlation was performed. *p* < 0.05 was considered significant. Blue dots indicate VR, and red dots indicate VNR. *indicates significant (*p* < 0.05) differences between VR versus VNR at indicated time points, with green star indicating higher levels in VRs compared to VNRs and grey star indicating higher levels in VNRs compared to VRs. Line indicates difference between time points within a group (green line VR; grey line VNR). *p < 0.05; **p < 0.01; ***p < 0.001. Underlying data used in the generation of this figure can be found in S2 Data. Ag.pTfh, antigen-specific peripheral T follicular helper; ICOS, inducible costimulator; VNR, vaccine nonresponder; VR, vaccine responder.
(TIF)

S3 Fig Higher inflammatory cytokines production in the PBMC culture supernatants from VNRs. PBMC culture supernatants obtained after the 5 d of H1N1 stimulation were subjected to IL-17, TNF-α, and activin A analysis by Magpix and ELISA. Dot plots showing levels of (C) IL-17, (D) TNF-α, and (E) activin A at T0 and T2 in VRs and VNRs from healthy (blue symbols) and HIV^+^ individuals (red symbols). For unpaired data, Mann-Whitney U test and for paired data Wilcoxon Signed Rank Test was performed. Error bar indicates the mean ± SD. *p* < 0.05 was considered significant. Blue dots indicate VR, and red dots indicate VNR. *p < 0.05; **p < 0.01; ***p < 0.001. Underlying data used in the generation of this figure can be found in S2 Data. IL-21, interleukin 21; PBMC, peripheral blood mononuclear cell; TNF-α, tumor necrosis factor alpha; VNR, vaccine nonresponder; VR, vaccine responder.
(TIF)

S4 Fig Single IL-21-producing CD40L+pTfh cells are higher in VR groups. PBMCs were stimulated with H1N1 antigen for 6 h in the presence of Brefeldin A. SEB was used as positive control and medium alone as negative control. Cells were stained for surface markers specific for pTfh cells along with live dead Aqua fixed, permeabilized and stained for intracellular cytokines (IL-2, IL-21, IFN-γ, and TNFα) and activation markers. CD40L^+^ pTfh cells were gated from central memory CD4 T cells and analyzed for the expression of different cytokines. (A) Functional combinations in CD40L^+^ pTfh cells were identified after Boolean gating. Pie chart represents 1, 2, 3, 4, and 5 functions, and bar chart shows all possible functional combinations at T0, T1, and T2. Data in the black box in the bar chart indicate the single IL-21-producing CD40L^+^pTfh cells at T0, T1, and T2. (B–E) Scatter plots showing (B) single IL-21^+^, (C) single TNFα^+^, (D) IL-21^+^TNFα^+^, and (E) IL-21^+^IL-2^+^TNFα^+^ CD40L^+^pTfh cells in VRs (green dots) and VNRs (grey dots). Line indicates difference between time points within a group (green line VR; grey line VNR). *p < 0.05; **p < 0.01; ***p < 0.001. Underlying data used in the generation of this figure can be found in S2 Data. IL-21, interleukin 21; PBMC, peripheral blood mononuclear cell; pTfh, peripheral T follicular helper; SEB, *Staphylococcus aureus* enterotoxin B; TNF-α, tumor necrosis factor alpha; VNR, vaccine nonresponder; VR, vaccine responder.
(TIF)

S5 Fig Association of Ag.pTfh frequency and IL-21^+^Ag.pTfh with B cell phenotypes post vaccination in HIV^+^ individuals and HCs. Correlation between H1N1-specific Ab-secreting MBCs at T2 with (A) IL-21^+^Ag.pTfh at T2, (B) TNFα^+^Ag.pTfh at T2. Correlation between (C) RM B cells at T2 with frequencies of Ag.pTfh cells at T2. For correlation analyses, Pearson correlation was performed based on data distribution. Error bar indicates the SEM. *p* < 0.05 was considered significant. HIV^+^ individuals are depicted in red dots and HCs in blue dots. Underlying data used in the generation of this figure can be found in S2 Data. Ag.pTfh, antigen-specific peripheral T follicular helper; HC, healthy control; IL-21, interleukin 21; MBC, memory B cell; RM, resting memory; TNFα, tumor necrosis factor alpha.
(TIF)

S6 Fig IL-21 Neutralization in APC:pTfh:MBC cocultures tend to lower the IgG production. pTfh, MBCs, and APCs collected at T2 from HIV^+^ (*n* = 3) and HC (*n* = 3) VRs were cultured with H1N1 Ag or SEB in the presence or absence of antihuman IL-21 neutralizing Ab for 7 d. Culture supernatants were harvested on day 7, and IgG production was measured by ELISA. H1N1-specific and SEB-specific IgG production in (A) HC VRs and (B) HIV^+^ VRs. Underlying data used in the generation of this figure can be found in S2 Data. Ag, antigen; APC, antigen presenting cell; HC, healthy control; MBC, memory B cell; pTfh, peripheral T follicular helper; SEB, *Staphylococcus aureus* enterotoxin B; VNR, vaccine nonresponder; VR, vaccine responder.
(TIF)

S7 Fig pTfh and MBC differentiation phenotypes in cocultures. PBMCs at T2 were thawed and rested overnight. pTfh (Aqua^−^CD19^−^CD3^+^CD8^−^CD4^+^CD45RO^+^CXCR5^+^), MBCs (Aqua^−^CD3^-^CD19^+^CD27^+^), and APC (Aqua^−^CD3^−^CD19^−^CD8^−^) populations were purified by cell sorting. B cells were cocultured with pTfh and APC at a 1:1:1 ratio in medium alone or in the presence of 5 μg/mL of H1N1 vaccine Ag +/− a cytokine cocktail containing IL-21 (50 ng/ml), anti-IL-2 (10 μg/ml), and antiTNF-α (2 μg/ml) or antiIL-21 neutralizing Ab (10 μg/ml) for 7 d. Harvested cells were analyzed for B cell differentiation and plasmablasts. Plasmablast differentiation of MBCs were identified as CD19^+^CD20^lo/neg^ and plasmablast as CD19^−^CD20^+^CD27^+^CD38^+^ for (A–B) cytokine cocktail conditions for VRs and VNRs and (C–D) IL-21 neutralization condition for VRs by flow cytometry. For unpaired data, Mann-Whitney U test and for paired data Wilcoxon signed rank test was performed. *p* < 0.05 was considered significant. Line with stars indicates difference between the conditions and groups and the level significance as *p < 0.05; **p < 0.01; ***p < 0.001. Underlying data used in the generation of this figure can be found in S2 Data. APC, antigen presenting cell; IL-21, interleukin 21; MBC, memory B cell; PBMC, peripheral blood mononuclear cell; pTfh, peripheral T follicular helper; TNFα, tumor necrosis factor alpha; VNR, vaccine nonresponder; VR, vaccine responder.
(TIF)

S1 Table Correlations between pTfh immune activation at T0 with Ag.pTfh frequency and function at T2. Spearman correlation was performed for correlation analysis, and *p* < 0.05 was considered significant. Correlation analysis included data from young and old VRs and VNRs together for HCs and HIV^+^ individuals. Ag.pTfh, antigen-specific pTfh; HC, healthy control; pTfh, peripheral T follicular helper; VNR, vaccine nonresponder; VR, vaccine responder.
(DOCX)

S2 Table Antigen-specific parameters at T0 and T2 included in the LASSO analysis. Symbols: “*,” Significant variables identified in LASSO analysis; “#,” variables common to HIV and HC; “$,” variables only in HC; “£,” variables only in HIV^+^ individuals. HC, healthy control; LASSO, least absolute shrinkage and selection operator.
(DOCX)

S1 Data Excel spreadsheet containing, in separate sheets, the underlying numerical data used in Figs 1, 2, 3, 4, 5, 6 and 7. (XLSX)

S2 Data Excel spreadsheet containing, in separate sheets, the underlying numerical data used in S1, S2, S3, S4, S5, S6 and S7 Figs. (XLSX)

## Abbreviations

Ab: antibody
Ag.pTfh: antigen-specific peripheral T follicular helper
AIM: activation-induced marker
APC: antigen presenting cell
ART: antiretroviral therapy
CCR4: CC chemokine receptor 4
CD: cluster of differentiation
CMV: Cytomegalovirus
CXCR5: CXC chemokine receptor 5
FC: fold change
FDA: Food and Drug Administration
FIND: Finding Novel Determinants of Flu Responses
FLORAH: Flu Responses of People in Relation to Age and HIV
GC: germinal center
GMT: geometric mean titer
HAI: hemagglutination inhibition
HC: healthy control
HIV+: HIV-infected persons
HLA-DR: Human Leukocyte Antigen–DR isotype
ICOS: inducible costimulator
ICOS-L: inducible costimulatory ligand
IL-21: interleukin 21
LASSO: least absolute shrinkage and selection operator
MBC: memory B cell
MoAb: monoclonal antibody
PBMC: peripheral blood mononuclear cell
PD-1: programmed cell death protein 1
PLSDA: partial least squares discriminant analysis
pTfh: peripheral T follicular helper
RM: resting memory
SEB: Staphylococcus aureus enterotoxin B
SPICE: Simplified Presentation of Incredibly Complex Evaluations
STAT5: Signal transducer and activator of transcription 5
Tfh: T follicular helper
Th17: T helper 17
TIV: trivalent influenza vaccine
TNFα: tumor necrosis factor alpha
VNR: vaccine nonresponder
VR: vaccine responder
