# Supplementary figures and images for: Dysfunctional peripheral T follicular helper cells dominate in people with impaired influenza vaccine responses: Results from the FLORAH study
Source: PLoS Biol. 2019 May 17;17(5):e3000257. doi: 10.1371/journal.pbio.3000257 (PMC6542545; doi:10.1371/journal.pbio.3000257)

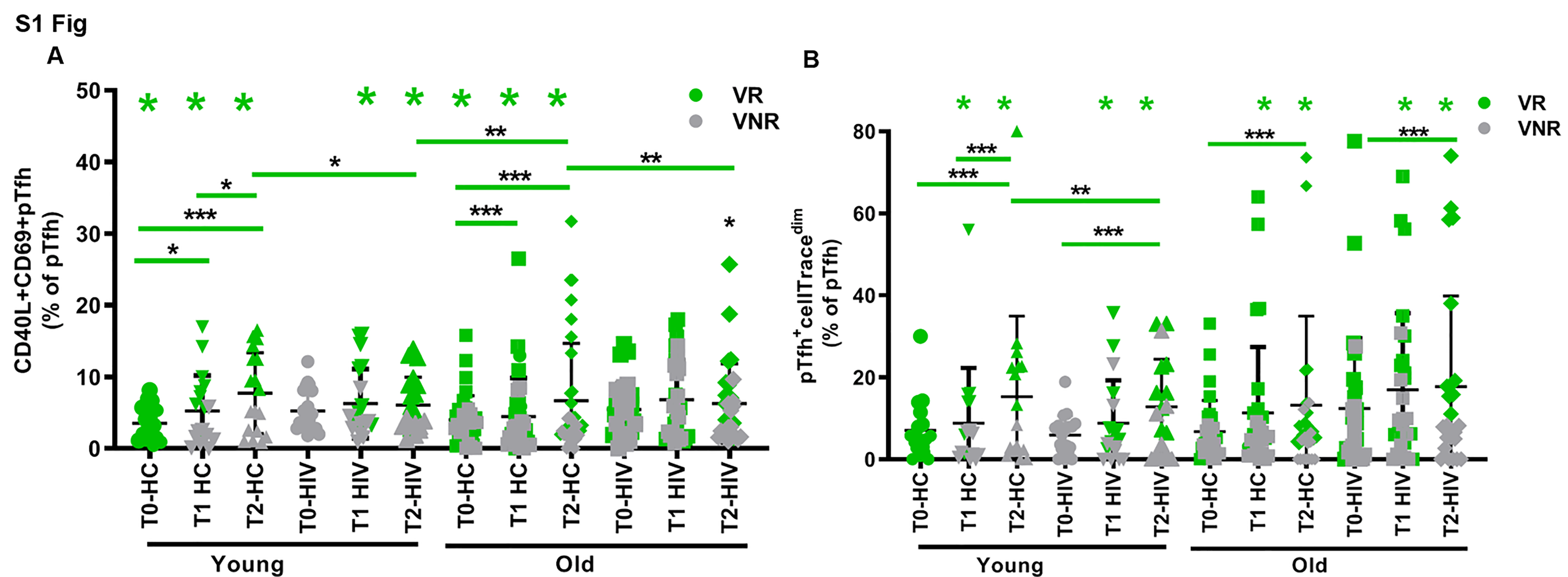

Supplement: S1 Fig — H1N1-specific pTfh cells were identified by flow cytometry in influenza-vaccinated young and old HIV+ individuals and HCs at baseline (T0), day 7 (T1), and day 28 (T2) post vaccination after 12 h of PBMC stimulation with H1N1 antigen. Proliferating pTfh cells were identified based on CellTrace dye dilution on day 5 after PBMC stimulation using flow cytometry. (A) Scatter plots showing frequencies of CD40L+CD69+ pTfh (Ag.pTfh) cells between different age groups in HIV+ individuals and HCs. (B), Scatter plots showing frequencies CellTracedimpTfh cells. Group and time analyses used generalized linear mixed models to accommodate the repeated measure of time for differences in the outcomes for each group separately between time points and also between 2 different groups at each time. Error lines indicates the mean ± SD. p < 0.05 was considered significant. *indicates significant (p < 0.05) differences between VR versus VNR at indicated time points, with green star indicating significantly higher in VRs compared to VNRs and lines with stars indicating difference between time points in VRs and VNRs (green line VR; grey line VNR). *p < 0.05; **p < 0.01; ***p < 0.001. Underlying data used in the generation of this figure can be found in S2 Data. Ag.pTfh, antigen-specific peripheral T follicular helper; HC, healthy control; PBMC, peripheral blood mononuclear cell; VNR, vaccine nonresponder; VR, vaccine responder. (TIF) [file pbio.3000257.s001.tif]

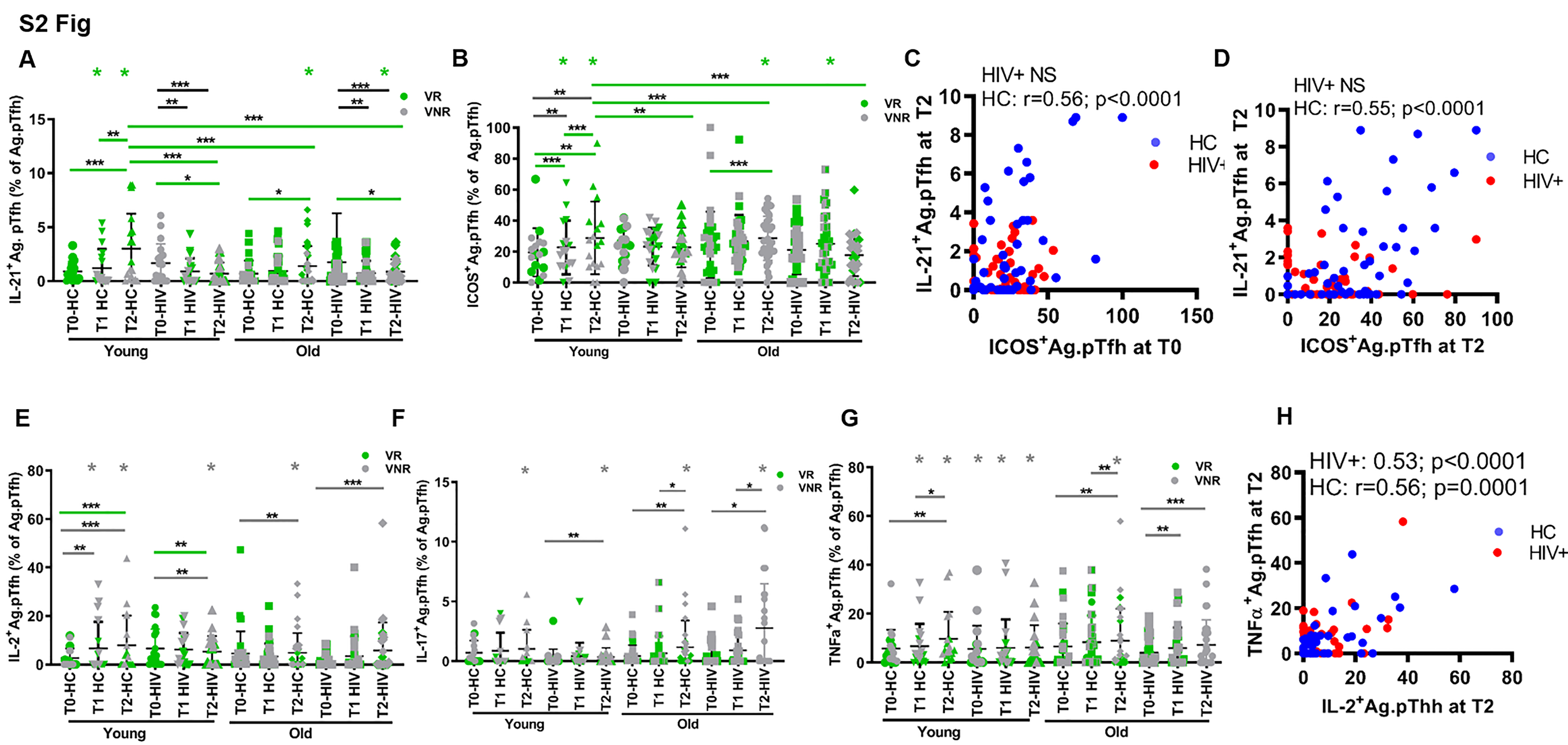

Supplement: S2 Fig — Scatter plots showing frequencies of (A) IL-21+Ag.pTfh cells, (B) ICOS+Ag.pTfh cells. (C–D) Correlation between IL-21+Ag.pTfh at T2 with ICOS+Ag.pTfh (C) at T0 and (D) at T2. Scatter plots showing frequencies of (E) IL-2+Ag.pTfh cells, (F) IL-17+Ag.pTfh cells, and (G) TNFα+Ag.pTfh cells. (H) Correlations between TNFα+Ag.pTfh at T2 with IL-2+Ag.pTfh at T2. Group and time analyses used generalized linear mixed models to accommodate the repeated measure of time for differences in the outcomes for each group separately between time points and also between 2 different groups at each time. Error lines indicates the mean ± SD. For correlation analyses, Pearson correlation was performed. p < 0.05 was considered significant. Blue dots indicate VR, and red dots indicate VNR. *indicates significant (p < 0.05) differences between VR versus VNR at indicated time points, with green star indicating higher levels in VRs compared to VNRs and grey star indicating higher levels in VNRs compared to VRs. Line indicates difference between time points within a group (green line VR; grey line VNR). *p < 0.05; **p < 0.01; ***p < 0.001. Underlying data used in the generation of this figure can be found in S2 Data. Ag.pTfh, antigen-specific peripheral T follicular helper; ICOS, inducible costimulator; VNR, vaccine nonresponder; VR, vaccine responder. (TIF) [file pbio.3000257.s002.tif]

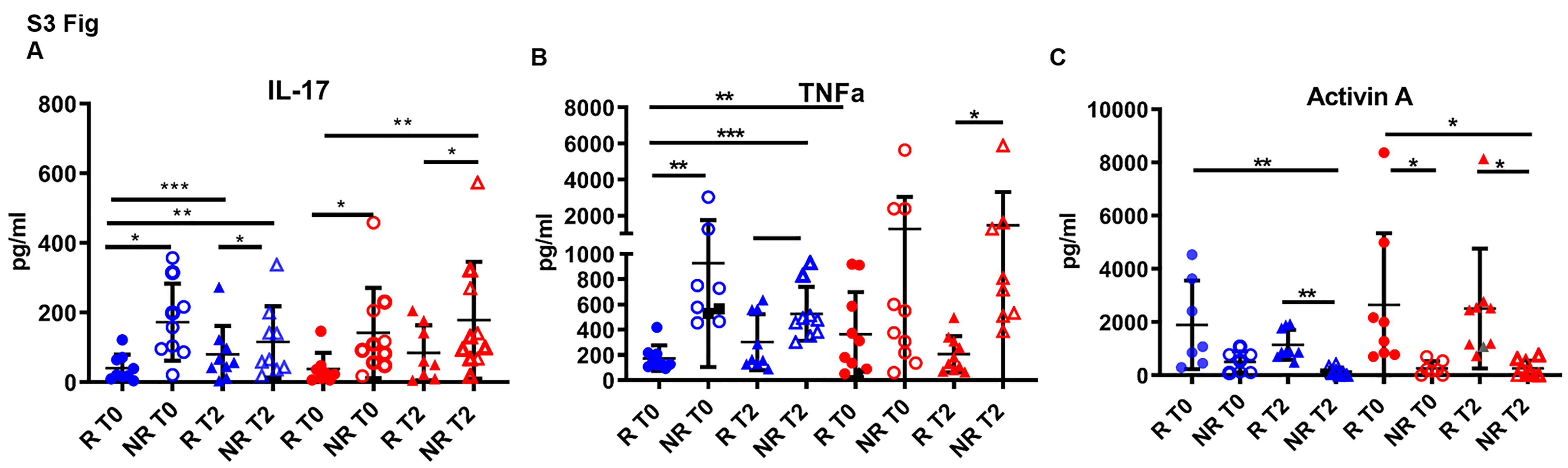

Supplement: S3 Fig — PBMC culture supernatants obtained after the 5 d of H1N1 stimulation were subjected to IL-17, TNF-α, and activin A analysis by Magpix and ELISA. Dot plots showing levels of (C) IL-17, (D) TNF-α, and (E) activin A at T0 and T2 in VRs and VNRs from healthy (blue symbols) and HIV+ individuals (red symbols). For unpaired data, Mann-Whitney U test and for paired data Wilcoxon Signed Rank Test was performed. Error bar indicates the mean ± SD. p < 0.05 was considered significant. Blue dots indicate VR, and red dots indicate VNR. *p < 0.05; **p < 0.01; ***p < 0.001. Underlying data used in the generation of this figure can be found in S2 Data. IL-21, interleukin 21; PBMC, peripheral blood mononuclear cell; TNF-α, tumor necrosis factor alpha; VNR, vaccine nonresponder; VR, vaccine responder. (TIF) [file pbio.3000257.s003.tif]

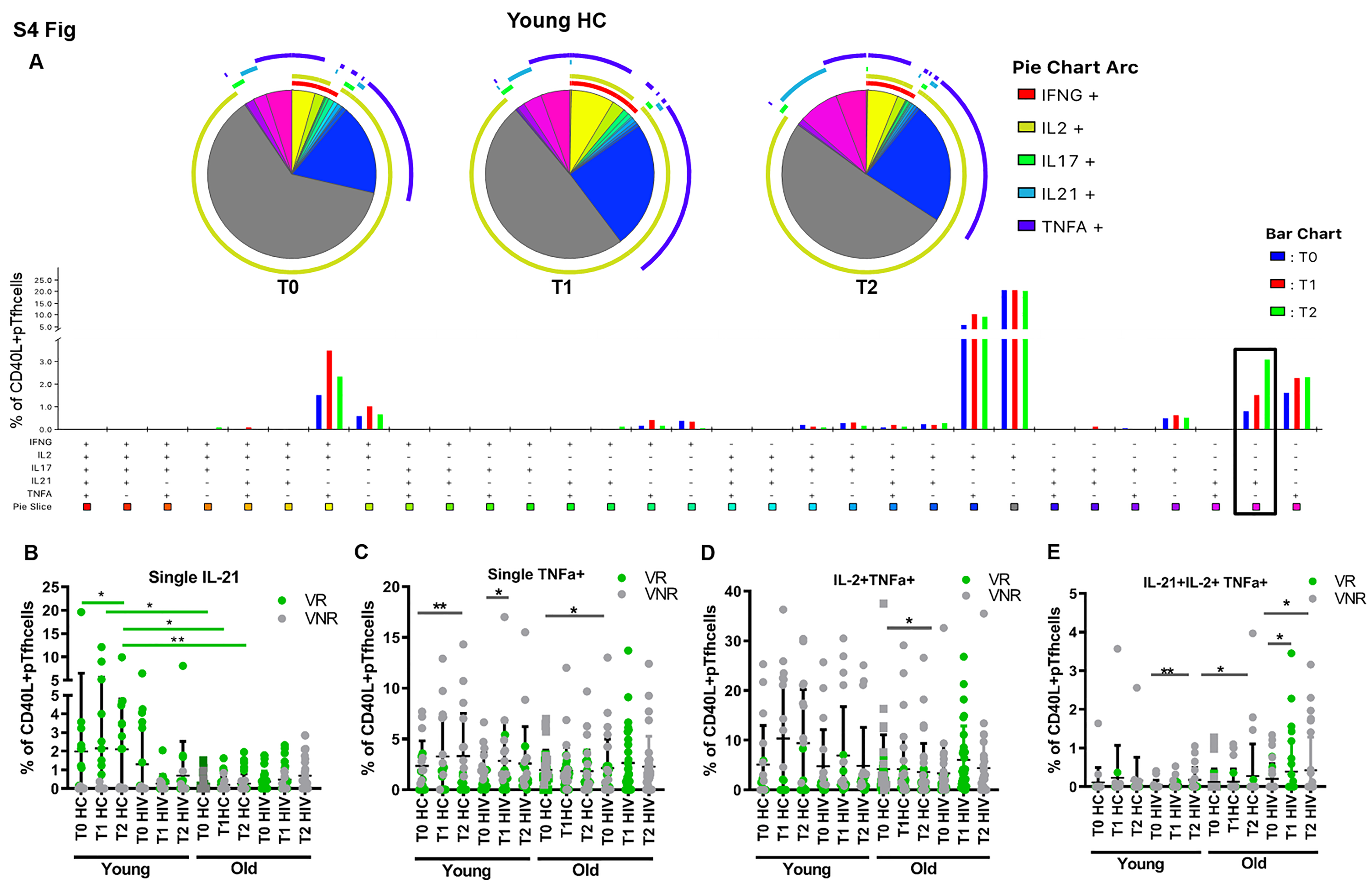

Supplement: S4 Fig — PBMCs were stimulated with H1N1 antigen for 6 h in the presence of Brefeldin A. SEB was used as positive control and medium alone as negative control. Cells were stained for surface markers specific for pTfh cells along with live dead Aqua fixed, permeabilized and stained for intracellular cytokines (IL-2, IL-21, IFN-γ, and TNFα) and activation markers. CD40L+ pTfh cells were gated from central memory CD4 T cells and analyzed for the expression of different cytokines. (A) Functional combinations in CD40L+ pTfh cells were identified after Boolean gating. Pie chart represents 1, 2, 3, 4, and 5 functions, and bar chart shows all possible functional combinations at T0, T1, and T2. Data in the black box in the bar chart indicate the single IL-21-producing CD40L+pTfh cells at T0, T1, and T2. (B–E) Scatter plots showing (B) single IL-21+, (C) single TNFα+, (D) IL-21+TNFα+, and (E) IL-21+IL-2+TNFα+ CD40L+pTfh cells in VRs (green dots) and VNRs (grey dots). Line indicates difference between time points within a group (green line VR; grey line VNR). *p < 0.05; **p < 0.01; ***p < 0.001. Underlying data used in the generation of this figure can be found in S2 Data. IL-21, interleukin 21; PBMC, peripheral blood mononuclear cell; pTfh, peripheral T follicular helper; SEB, Staphylococcus aureus enterotoxin B; TNF-α, tumor necrosis factor alpha; VNR, vaccine nonresponder; VR, vaccine responder. (TIF) [file pbio.3000257.s004.tif]

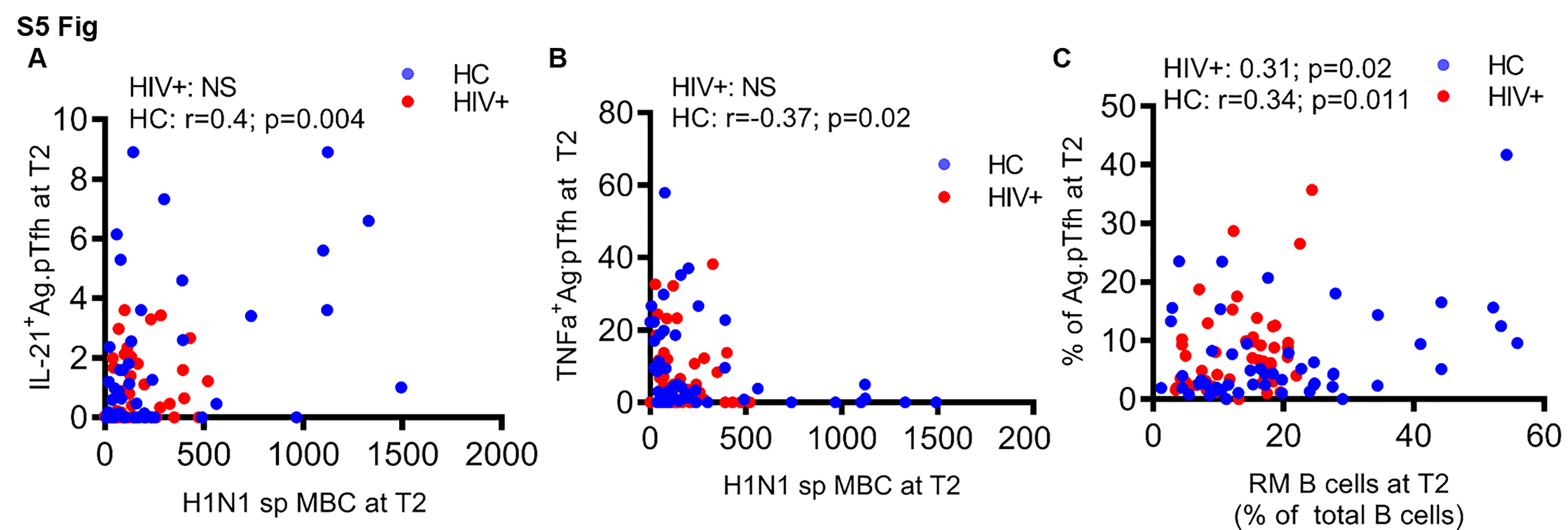

Supplement: S5 Fig — Correlation between H1N1-specific Ab-secreting MBCs at T2 with (A) IL-21+Ag.pTfh at T2, (B) TNFα+Ag.pTfh at T2. Correlation between (C) RM B cells at T2 with frequencies of Ag.pTfh cells at T2. For correlation analyses, Pearson correlation was performed based on data distribution. Error bar indicates the SEM. p < 0.05 was considered significant. HIV+ individuals are depicted in red dots and HCs in blue dots. Underlying data used in the generation of this figure can be found in S2 Data. Ag.pTfh, antigen-specific peripheral T follicular helper; HC, healthy control; IL-21, interleukin 21; MBC, memory B cell; RM, resting memory; TNFα, tumor necrosis factor alpha. (TIF) [file pbio.3000257.s005.tif]

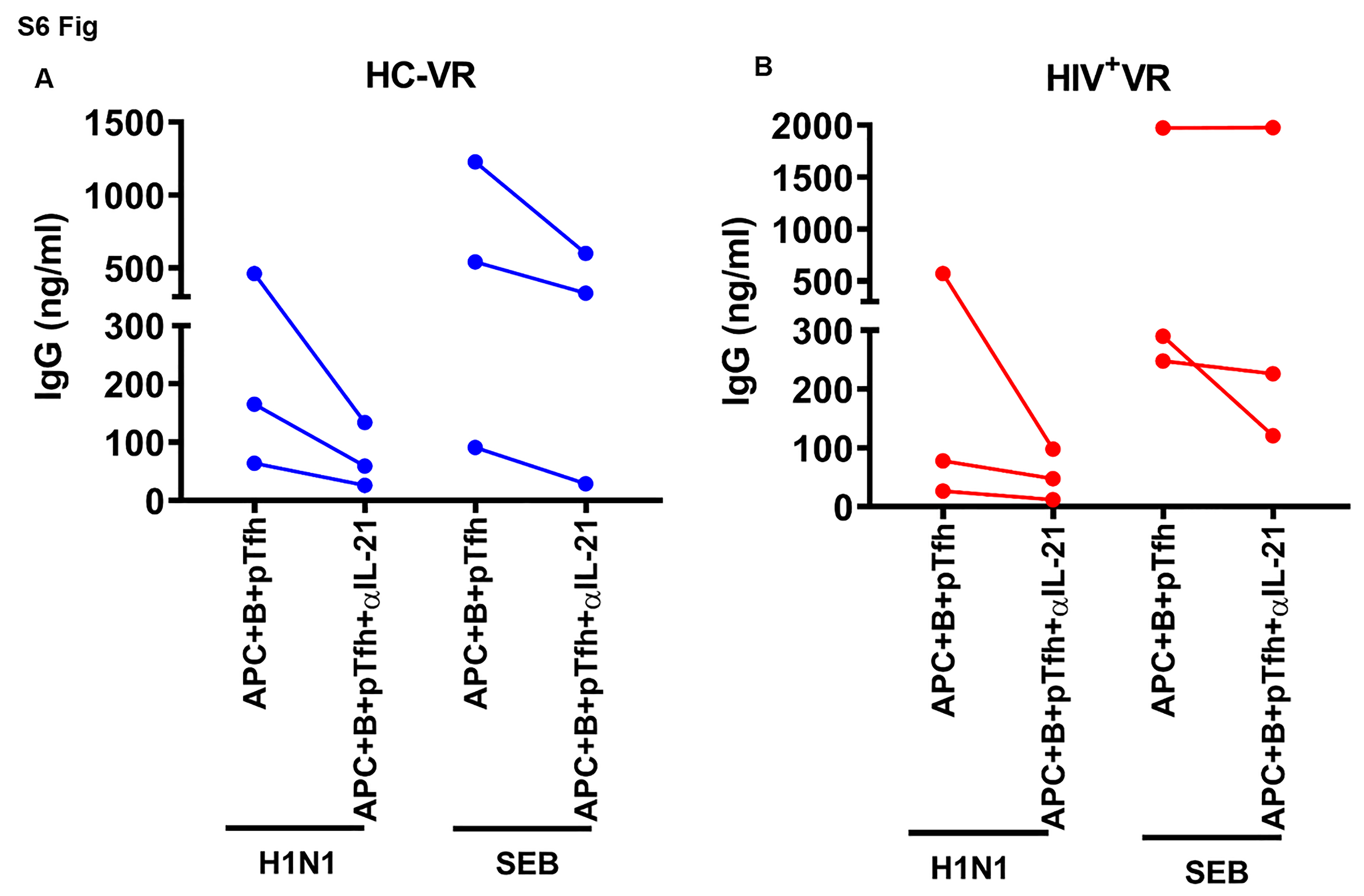

Supplement: S6 Fig — pTfh, MBCs, and APCs collected at T2 from HIV+ (n = 3) and HC (n = 3) VRs were cultured with H1N1 Ag or SEB in the presence or absence of antihuman IL-21 neutralizing Ab for 7 d. Culture supernatants were harvested on day 7, and IgG production was measured by ELISA. H1N1-specific and SEB-specific IgG production in (A) HC VRs and (B) HIV+ VRs. Underlying data used in the generation of this figure can be found in S2 Data. Ag, antigen; APC, antigen presenting cell; HC, healthy control; MBC, memory B cell; pTfh, peripheral T follicular helper; SEB, Staphylococcus aureus enterotoxin B; VNR, vaccine nonresponder; VR, vaccine responder. (TIF) [file pbio.3000257.s006.tif]

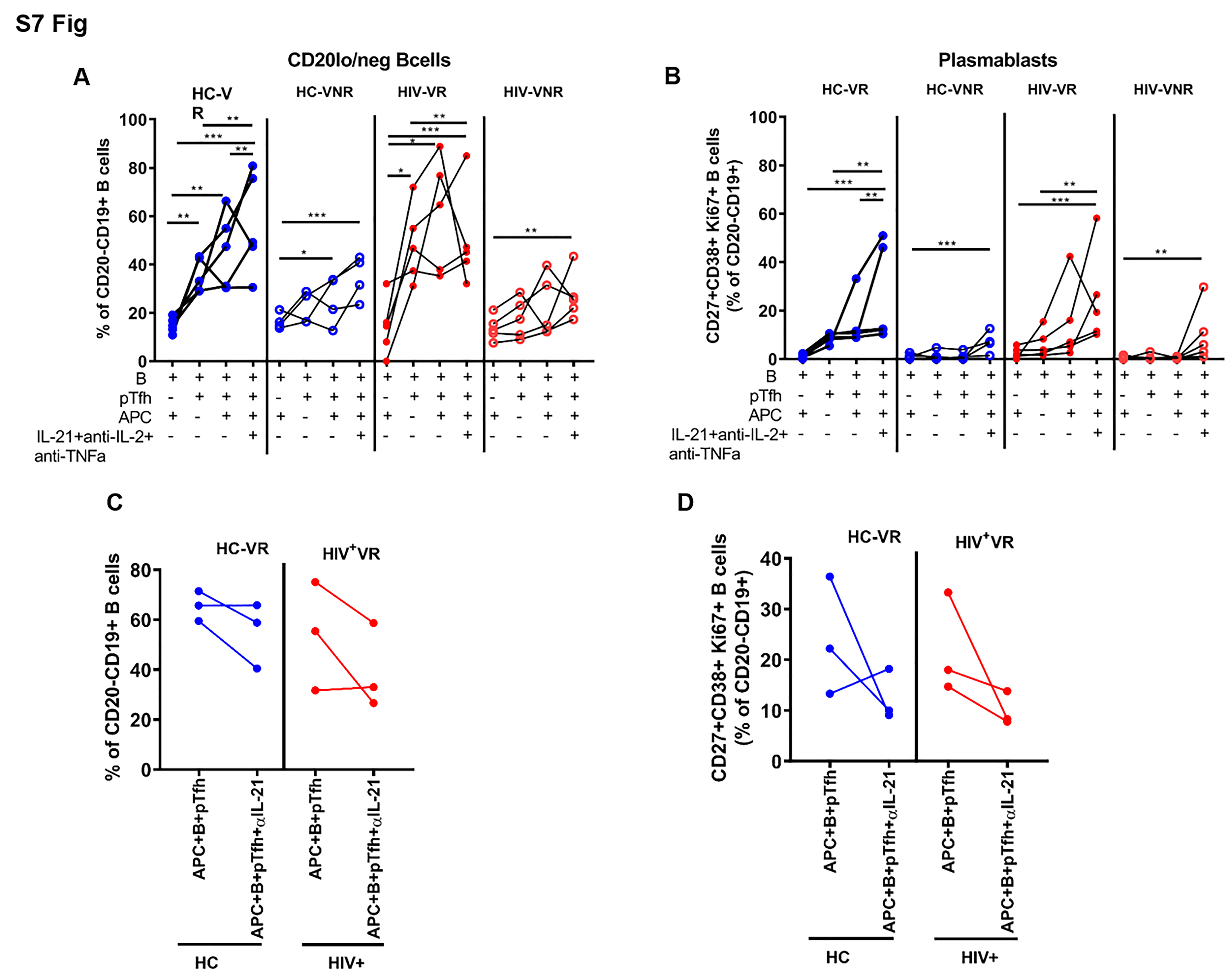

Supplement: S7 Fig — PBMCs at T2 were thawed and rested overnight. pTfh (Aqua−CD19−CD3+CD8−CD4+CD45RO+CXCR5+), MBCs (Aqua−CD3-CD19+CD27+), and APC (Aqua−CD3−CD19−CD8−) populations were purified by cell sorting. B cells were cocultured with pTfh and APC at a 1:1:1 ratio in medium alone or in the presence of 5 μg/mL of H1N1 vaccine Ag +/− a cytokine cocktail containing IL-21 (50 ng/ml), anti-IL-2 (10 μg/ml), and antiTNF-α (2 μg/ml) or antiIL-21 neutralizing Ab (10 μg/ml) for 7 d. Harvested cells were analyzed for B cell differentiation and plasmablasts. Plasmablast differentiation of MBCs were identified as CD19+CD20lo/neg and plasmablast as CD19−CD20+CD27+CD38+ for (A–B) cytokine cocktail conditions for VRs and VNRs and (C–D) IL-21 neutralization condition for VRs by flow cytometry. For unpaired data, Mann-Whitney U test and for paired data Wilcoxon signed rank test was performed. p < 0.05 was considered significant. Line with stars indicates difference between the conditions and groups and the level significance as *p < 0.05; **p < 0.01; ***p < 0.001. Underlying data used in the generation of this figure can be found in S2 Data. APC, antigen presenting cell; IL-21, interleukin 21; MBC, memory B cell; PBMC, peripheral blood mononuclear cell; pTfh, peripheral T follicular helper; TNFα, tumor necrosis factor alpha; VNR, vaccine nonresponder; VR, vaccine responder. (TIF) [file pbio.3000257.s007.tif]
