# Supplementary material for: Dysfunctional peripheral T follicular helper cells dominate in people with impaired influenza vaccine responses: Results from the FLORAH study
Source: PLoS Biol. 2019 May 17;17(5):e3000257. doi: 10.1371/journal.pbio.3000257 (PMC6542545; doi:10.1371/journal.pbio.3000257)
Supplement: S1 Table — Spearman correlation was performed for correlation analysis, and p < 0.05 was considered significant. Correlation analysis included data from young and old VRs and VNRs together for HCs and HIV+ individuals. Ag.pTfh, antigen-specific pTfh; HC, healthy control; pTfh, peripheral T follicular helper; VNR, vaccine nonresponder; VR, vaccine responder. (DOCX) [file pbio.3000257.s008.docx]

**S1 Table: Correlations between pTfh immune activation at T0 with Ag.pTfh frequency and function at T2**

|  | **HC** | | **HIV+** | |
| --- | --- | --- | --- | --- |
| **pTfh: DR+CD38+ at T0 vs.** | **p** | **r** | **p** | **r** |
| Ag.pTfh at T2 (%) | 0.232 | -0.179 | 0.649 | 0.067 |
| ICOS+Ag.pTfh at T2 | **0.005** | **-0.403** | **0.023** | **-0.36** |
| IL-21+Ag.pTfh at T2 | 0.081 | -0.206 | 0.120 | -0.13 |
| IL-2+Ag.pTfh at T2 | 0.245 | 0.174 | 0.343 | 0.139 |
| IL-17+Ag.pTfh at T2 | 0.167 | 0.207 | 0.387 | 0.127 |
| TNFa+Ag.pTfh at T2 | **0.016** | **0.324** | **0.009** | **0.391** |
|  |  |  |  |  |
| **pTfh: DR+38+PD1+ at T0 Vs.** |  |  |  |  |
| Ag.pTfh at T2 (%) | **0.026** | **-0.366** | **0.014** | **-0.39** |
| ICOS+Ag.pTfh at T2 | 0.086 | -0.184 | **0.002** | **-0.4** |
| IL-21+Ag.pTfh at T2 | 0.303 | -0.09 | 0.148 | -0.067 |
| IL-2+Ag.pTfh at T2 | 0.792 | 0.039 | 0.275 | 0.166 |
| IL-17+Ag.pTfh at T2 | 0.142 | 0.219 | 0.714 | 0.054 |
| TNFa+Ag.pTfh at T2 | **0.012** | **0.354** | **0.001** | **0.464** |
