# Supplementary material for: Dysfunctional peripheral T follicular helper cells dominate in people with impaired influenza vaccine responses: Results from the FLORAH study
Source: PLoS Biol. 2019 May 17;17(5):e3000257. doi: 10.1371/journal.pbio.3000257 (PMC6542545; doi:10.1371/journal.pbio.3000257)
Supplement: S2 Table — Symbols: “*,” Significant variables identified in LASSO analysis; “#,” variables common to HIV and HC; “$,” variables only in HC; “£,” variables only in HIV+ individuals. HC, healthy control; LASSO, least absolute shrinkage and selection operator. (DOCX) [file pbio.3000257.s009.docx]

**S2 Table: Antigen specific parameters at T0 and T2 included in the LASSO analysis**

| 1. H1N1stimBulkpTfh [(% of CD45RO+CD27+CD4): CXCR5 expressing central memory CD4 T cells after H1N1 stimulation)] |
| --- |
| 1. CD4CD40L [(% of CD4); CD40L expressing CD4 T cells] |
| 1. CD40LpTfh [(% of CD40L): pTfh cells within CD40L+CD4 T cells]*$ |
| 1. pTfhCD40L [(% of pTfh): CD40L expressing pTfh cells)] |
| 1. CD40LpTfhIL21 (% of CD40LpTfh) |
| 1. pTfhCD40LIL21 (% of pTfhCD40L) |
| 1. CD40LpTfhIL2 (% of CD40LpTfh) |
| 1. pTfhCD40LIL2 (% of pTfhCD40L) |
| 1. pTfhCD40LTNFα (% of pTfhCD40L) |
| 1. pTfhCD40LIL17 (% of pTfhCD40L)*$ |
| 1. CD4IL21(% of CD4) |
| 1. CD4IL2 (% of CD4) |
| 1. CD4TNFα(% of CD4) |
| 1. CD4IL17 (% of CD4) |
| 1. CD4ILIFNg (% of CD4) |
| 1. BulkpTfh [(% of CD45ROCD4); CXCR5+ central memory CD4] |
| 1. CD4CD40LCD69 [(% of CD4)*; CD40Land CD69 dual expressing CD4]*# |
| 1. CD40LCD69pTfh* [(% of CD40LCD69); CD40Land CD69 dual expressing pTfh (Ag-pTfh)]*# |
| 1. CD40LCD69pTfhICOS[ (% of CD40LCD69pTfh); Ag-pTfh ICOS] |
| 1. CD40LCD69pTfhIL21 [(% of CD40LCD69pTfh*); IL-21+Ag-pTfh]*$ |
| 1. CD40LCD69pTfhIL2 (% of CD40LCD69pTfh*); IL-2+Ag-pTfh]* |
| 1. CD40LCD69pTfhIFNg [(% of CD40LCD69pTfh);IFNg+Ag-pTfh] |
| 1. CD40LCD69pTfhIL17 [(% of CD40LCD69pTfh); IL-17+Ag-pTfh] |
| 1. CD40LCD69pTfhTNFα [(% of CD40LCD69pTfh); TNFα+Ag-pTfh] |
| 1. CD4CellTrace [(% of CD4): CellTrace^dim^ CD4 T cells)] |
| 1. pTfhCelltrace [(% of pTfh)*; CellTrace^dim^ pTfh cells)]*£ |
| 1. pTfhCelltraceICOS [(% of pTfhCellTrace) CellTrace^dim^ pTfh expressing ICOS] |
